# Systematic review of cigar smoking and all cause and smoking related mortality

**DOI:** 10.1186/s12889-015-1617-5

**Published:** 2015-04-24

**Authors:** Cindy M Chang, Catherine G Corey, Brian L Rostron, Benjamin J Apelberg

**Affiliations:** Office of Science, Center for Tobacco Products, Food and Drug Administration, Document Control Center, Building 71, Room G335, 10903 ,New Hampshire Avenue, Silver Spring, MD 20993-0002 USA

**Keywords:** Cigars, Mortality, Systematic review

## Abstract

**Background:**

Cigars are a growing public health concern, given the changes in cigar use patterns in the US and elsewhere since the 1960s. We conducted a systematic review of published studies on current cigar smoking and all-cause and cause-specific mortality risks to inform potential regulatory approaches and future research that would strengthen the body of evidence.

**Methods:**

Using 3 different databases and handsearching, we identified epidemiological studies published prior to June 2014 that examined the association between cigar smoking and all-cause mortality and smoking-related mortality. Detailed study characteristics as well as association-level characteristics, including effect estimates and 95% confidence intervals, were abstracted or calculated from each selected study.

**Results:**

A total of 22 studies from 16 different prospective cohorts were identified. Primary cigar smoking (current, exclusive cigar smoking with no history of previous cigarette or pipe smoking) was associated with all cause-mortality, oral cancer, esophageal cancer, pancreatic cancer, laryngeal cancer, lung cancer, coronary heart disease (CHD), and aortic aneurysm. Strong dose trends by cigars per day and inhalation level for primary cigar smoking were observed for oral, esophageal, laryngeal, and lung cancers. Among primary cigar smokers reporting no inhalation, relative mortality risk was still highly elevated for oral, esophageal, and laryngeal cancers.

**Conclusions:**

In summary, cigar smoking carries many of the same health risks as cigarette smoking. Mortality risks from cigar smoking vary by level of exposure as measured by cigars per day and inhalation level and can be as high as or exceed those of cigarette smoking. The body of evidence would be strengthened by future studies that focus on the health effects of primary cigar smoking and incorporate more contemporary and diverse study populations to better reflect the current patterns of cigar use in the US. Ideally, these studies would also collect detailed information on cigar type, exposure level, and biomarkers of exposure and potential harm.

**Electronic supplementary material:**

The online version of this article (doi:10.1186/s12889-015-1617-5) contains supplementary material, which is available to authorized users.

## Background

The 2009 Family Smoking Prevention and Tobacco Control Act (FSPTCA) provided the US Food and Drug Administration (FDA) with regulatory authority over the manufacture, distribution, and marketing of cigarettes, roll-your-own tobacco, and smokeless tobacco. In April 2014, the FDA issued a proposed rule to assert jurisdiction over additional tobacco products, including cigars which are a growing public health concern [1]. The US Centers for Disease Control and Prevention (CDC) has reported that from 2000 to 2011, cigar consumption more than doubled in the US from slightly less than 6.2 billion sticks in 2000 to more than 13.7 billion in 2011 [2]. Cigarette consumption, on the other hand, declined by 33% in the US during this period. In the US, the current cigar product landscape varies widely with respect to flavors, pack sizes, brands and cigar types (e.g., large cigars, cigarillos, and little filtered cigars) [3]. Cigar use among youth and young adults has been of particular concern in recent years. Nearly 16% of US young adults ages 18 to 24 years reported having smoked cigars on at least one day in the past 30 days in 2009–2010 [4]. Nationally, 2.8% of middle school students and 12.6% of high school students reported having smoked cigars (either cigars, cigarillos or little cigars) on at least one day in the past 30 days [5]. In the same study, cigar smoking prevalence significantly increased from 2011 to 2012 in non-Hispanic Black high school students from 11.7% to 16.7%.

Cigar smoking produces numerous adverse health effects. Cigar smoke contains many of the same toxic constituents as cigarette smoke, and cigar smoke has been shown to have higher levels of tobacco-specific nitrosamines (TSNAs) than cigarette smoke due to the curing and fermentation process for cigar tobacco [6]. Many of these TSNAs such as N-Nitrosonornicotine (NNN) and nicotine-derived nitrosamine ketone (NNK) are known carcinogens [6]. Cigar smoke has also been found to have higher levels of carbon monoxide and nitrogen oxide than cigarette smoke [6]. The International Agency of Research on Cancer (IARC) has previously concluded in 2004 that cigar and/or pipe smoking is causally connected to cancers of the lung and upper aerodigestive tract, including the oral cavity, oropharynx, hypopharynx, larynx and esophagus. They also found evidence that cigar and/or pipe smoking are causally associated with cancers of the pancreas, stomach and urinary bladder [7,8]. A recent analysis of the population health effects of cigar smoking found that regular cigar smoking is responsible for at least 9,000 deaths each year in the US and that the total number of deaths may be higher due to potential increases in cigar smoking relative risks over time, perhaps due to more diverse cigar products and changes in inhalation patterns, as well as the possibility of deaths due to other causes that have not been previously linked to cigar smoking [9].

Given the public health and regulatory importance of cigars, it is extremely timely and important to have accurate and comprehensive information on the health effects of these products. This study presents a systematic review of published studies on current cigar smoking and all-cause and cause-specific mortality risks. In doing so, it synthesizes the information currently available on the subject and identifies areas for further research.

## Methods

We conducted a systematic review of epidemiological studies published prior to June 2014 that examined the association between cigar smoking and all-cause and cause-specific mortality. In doing so, we followed guidelines for systematic reviews from the Institute of Medicine (IOM) [10], the Meta-analysis Of Observational Studies in Epidemiology (MOOSE) group [11] and the Cochrane Collaboration [12]. For cause-specific mortality, we selected causes and conditions identified as smoking-related in the 2004 Surgeon General’s Report on the health effects of smoking and IARC’s 2012 summary monograph “A Review of Human Carcinogens” [13,14]. Based on these criteria, studies of cigar smoking and the following causes of death were identified in the research literature and included in this review: oral cancer, nasopharyngeal carcinoma, esophageal cancer, stomach cancer, colon and rectal cancer, liver cancer, pancreatic cancer, laryngeal cancer, lung cancer, bladder cancer, kidney cancer, atherosclerosis, coronary heart disease, stroke, aortic aneurysm, and chronic obstructive pulmonary disease (COPD).

We conducted a search in PubMed using the following terms: (cigar[tw] OR cigars[tw] OR cigarillo[tw] OR cigarillos[tw] OR cheroot[tw] OR cheroots[tw] OR stogies[tw]) AND (death[tw] OR mortality[tw]), yielding 100 potentially pertinent references. We also conducted a search in EMBASE using the following terms: (cigar OR cigars OR cigarillo OR cigarillos OR cheroot OR cheroots OR stogies) AND (‘death’/exp OR death OR ‘mortality’/exp OR mortality), yielding 207 potential references. Finally, we searched ISI Web of Science using the terms: Topic = (cigar OR cigars OR cigarillo OR cigarillos OR cheroot OR cheroots OR stogies) AND Topic = (death OR mortality), yielding 92 potential references. After removing duplicate records among the three search results, there were a total of 246 references.

### Study selection

**Figure 1:**
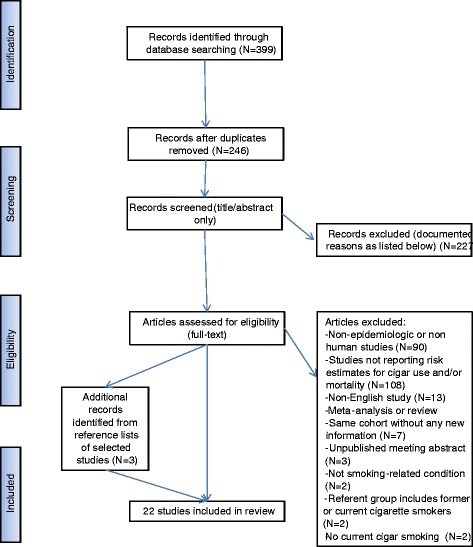
**Flowchart of study selection of prospective studies of cigar and all-cause and cause-specific mortality.**

At each stage of the study screening, two reviewers (CMC and CGC) independently reviewed the studies and made selections for inclusion (Figure 1). Final selections were made after discussion and consensus was reached on discrepant results. All selected studies were screened by title and abstract, and the full texts of the subset of relevant papers were then reviewed. A total of 227 references were excluded from review (see Additional file 1).

Because the focus of this review is on studies that examine current cigar smoking at baseline, we excluded an earlier cohort study conducted by the American Cancer Society that looked at mortality risks for lifetime ever cigar smokers (w1-w2). A total of 108 references did not report mortality risk associated with cigar use (w3-w110). An additional 13 non-English studies were also excluded (w111-w123). We excluded 90 non-epidemiologic or non-human studies, including reviews and commentaries (w124-w213). Two studies were excluded because they examined causes of death (prostate cancer and multiple myeloma) that were not classified as smoking-related in the 2004 Surgeon General’s Report or 2012 IARC monograph (w214-w215). Because the aim of this review was to compare mortality risks relative to never tobacco users or never smokers, two studies with former or current cigarette smokers in their reference groups were excluded (w216-w217). We excluded 7 references because they only contained results from cohort studies that were also published in studies that were selected for review (w218-w224). Three references were excluded because they were unpublished meeting abstracts (w225-w227). Finally, three additional references were identified by handsearching the references of relevant studies. In total, 22 articles on cigar smoking and all-cause and cause-specific mortality were included in this review [15-36] (Figure 1 and Table 1).

**Table 1:** **Selected prospective studies of cigar smoking and mortality**

**Author**	**Publication year**	**Country**	**Cohort name**	**Follow-up**		**Total participants**	**Total current cigar and/pipe smokers**	**Age range**	**Effect measure reported**
**Kahn** [28]	1966	US	Dorn study	1954-1957	to 1962	248,195	N/A	31 to 84	SMR
**Best** [15]	1966	Canada	Canadian Study of Smoking and Health	1956	to 1962	92,000	1,594	30 to 90	SMR
**Cole** [19]	1974	Denmark		1958	to 1972	183	N/A	55 to 64	Calculated IRR
**Gordon** [36]	1974	US	Framingham	1948-1953	to 1966-1970	2,336	311	29 to 62	Calculated IRR
**Doll** [22]	1976	UK	Male British Doctors	1951	to 1971	34,440	N/A	20 and older	Calculated SMR
**Jajich** [27]	1984	US	Cook County, IL	1965	to 1970	2,674	265 (18 females)	65 to 74	IRR
**Carstensen** [17]	1987	Sweden	Swedish Census Cohort	1963	to 1979	25,129	N/A	18 to 69	SMR
**Sandler** [29]	1989	US	Washington, County, MD	1963	to 1975	46,926	1,671 (10 females)	25 and older	IRR, 95% CI
**Hsing** [25]	1990	US	Dorn study	1954-1957	to 1980	249,829	N/A	31 to 84	IRR, 95% CI
**Strachan** [34]	1991	UK	Nested case–control within Whitehall study cohort	1967-1969	to 1987	18,403	N/A	40 to 64 years	OR, 95% CI
**Lange** [30]	1992	Denmark	Copenhagen City Heart Study	1976	to 1989	14,214	808 (770 females)	20 and older	HR, 95% CI
**Chow** [21]	1992	US	Lutheran Brotherhood Insurance Society	1966	to 1986	17,818	N/A	35 years and older	IRR, 95% CI
**Chow** [20]	1993	US	Dorn study	1954-1957	to 1980	248,046	N/A	31 to 84	IRR, 95% CI
**Ben-Shlomo** [16]	1994	UK	Whitehall study	1967-1969	to 1987	19,018	763	40 to 69	Calculated IRR
**Heineman** [24]	1995	US	Dorn study	1954-1957	to 1980	248,046	N/A	31 to 84	IRR, 95% CI
**Haheim** [23]	1996	Finland	Oslo study	1972	to 1992	16,173	1,623	40 to 49	HR, 95% CI
**Wald** [35]	1997	UK	British United Provident Association	1975-1982	to 1993	21,520	1,831	35 to 64	HR, 95% CI
**Shanks** [31]	1998	US	CPS-I	1959-1960	to 1972	442,455	15,191 (primary)	30 and older	IRR, 95% CI
**Jacobs** [26]	1999	US	CPS-II	1982	to 1991	121,278	6,914	30 years or older	HR, 95% CI
**Shapiro** [33]	2000	US	CPS-II	1982	to 1994	508,353	7,888	30 years or older	HR, 95% CI
**Chao** [18]	2002	US	CPS-II	1982	to 1996	467,788	6,945	30 years or older	HR, 95% CI
**Shaper** [32]	2003	UK	British Regional Heart Study	1978-1989	to 2000	7,121	730	40 to 59	HR, 95% CI

### Data extraction

Two different sets of data were extracted. The first dataset consisted of study-level characteristics including cohort name, country where study was conducted, publication year of study, number of cigar users, age range of cohort participants, year of enrollment, length of follow-up, outcome type (i.e. all-cause or cause-specific mortality), and relative mortality risk measure. The second dataset consisted of association-level characteristics including relative risk estimates as well as characteristics used to stratify estimates such as age, type and level of cigar exposure, and duration of cigar exposure. Thus, multiple entries could occur for a particular study. Additionally, reported International Classification of Diseases (ICD) codes from the relevant revision were extracted for cause-specific mortality estimates. Although one included study, Shanks and Burns (1998), did not report ICD codes in their analysis of Cancer Prevention Study I (CPS-I) data, it can be assumed that their cause of death coding system was similar to ICD 7, given that similar studies of CPS-I have used ICD 7 codes [37]. Data extraction was independently conducted by two reviewers (CMC and CGC). Similar to the selection of studies, data entries were compared and any differences in data extraction were resolved through discussion.

A number of studies did not report 95% confidence intervals and/or relative mortality risks (see “Effect measure reported” column in Table 1). For these studies, we calculated these estimates using the available data. For standardized mortality ratios (SMRs), we used the relevant formula from Breslow et al. (equation 2.15) to calculate 95% confidence intervals [38]. For incidence rate ratios (IRRs), we calculated 95% confidence intervals using Wald Limits [39].

The main exposure of interest was either current exclusive cigar smoking or current exclusive cigar/pipe smoking. Current cigarette smokers are excluded from this analysis to better isolate the effects of cigar smoking on disease risk. Past cigarette smoking patterns are likely to affect current cigar smoking patterns. CPS-I results suggest that secondary cigar smokers (current, exclusive cigar smoking with a history of previous cigarette or pipe smoking) are about twice as likely to report inhaling cigar smoke as primary cigar smokers (current, exclusive cigar smoking with no history of previous cigarette or pipe smoking) (42.0% vs. 21.6%) [31]. Therefore, secondary cigar smokers may have different disease risks compared with primary cigar smokers not only due to past cigarette use, but also due to differences in cigar use, particularly with inhalation. Thus, for studies that assessed this information, we present results for primary and secondary cigar smokers separately as well as separate studies that combine cigar and pipe use from studies of cigar use only.

## Results

In total, there were 22 selected studies that examined cigar smoking and mortality from 16 cohorts (Table 1). All of the studies were from prospective cohorts for which vital status was usually determined through either active follow-up or linkage to a death registry (Strachan conducted a case–control study nested within a prospective cohort [34]). Four studies were published from the Dorn study cohort which approximately 250,000 government life-insurance policy holders (mostly US World War I veterans) responded to questionnaires on tobacco use mailed to them in the 1950s. Two studies were published from the Whitehall study cohort in which over 18,000 men aged 40–69 years from the British Civil Service underwent an examination between 1967 and 1969 which included reporting health history and lifetime smoking habits. Finally, three studies were published from the American Cancer Society’s Cancer Prevention Study II (CPS-II) cohort in which over a half million male volunteers from all 50 US states completed a self-administered questionnaire in 1982. Women were not included in CPS-II studies of cigar use because they were not asked if they smoked cigars. One of the 16 cohorts began in the 1940s, five cohorts began in the 1950s, five cohorts began in the 1960s, four cohorts began in the 1970s, and one cohort began in the 1980s. The studies were conducted primarily in North America and Europe with 12 in the US, five in the United Kingdom (UK), one in Canada, and four in Nordic countries (Denmark, Sweden, and Finland).

We found a substantial amount of variation across studies in terms of study characteristics including the definition of tobacco exposure (e.g. assessment of past cigarette smoking, inclusion of current pipe smoking), dose categories, and adjustment for possible confounding risk factors. Furthermore, CPS-I and II were the only cohorts large enough to yield results for primary cigar smokers and secondary cigar smokers separately for most outcomes. Thus, we present the results descriptively, rather than giving pooled relative risk estimates. In the subsequent results, we refer to effect estimates as “mortality ratios” or MRs, regardless of whether they are age-standardized mortality ratios, hazards ratios (HR), incidence rate ratios (IRR) or odds ratios (OR). We present MRs for current cigar smoking only (rather than ever or former cigar smoking). The referent group is never smokers or never tobacco users. Results for all-cause mortality and cause-specific mortality are presented. Estimates for groups of causes, such as cancers of the digestive system or genitourinary system, were not included in our results. For each outcome, we present results in the following order of exposure: primary cigar smoking (current, exclusive cigar smoking with no previous history of cigarette or pipe smoking), primary cigar and/or pipe smoking (current, exclusive cigar and/or pipe smoking with no previous history of cigarette smoking), secondary cigar smoking (current, exclusive cigar smoking with previous history of cigarette or pipe smoking), secondary cigar and/or pipe smoking (current, exclusive cigar and/or pipe smoking with previous history of cigarette smoking), any current cigar smoking (regardless of cigarette/pipe smoking history), and any current cigar/pipe smoking (regardless of cigarette smoking history). Following overall cigar exposure, we then present results (when available) by level of cigar exposure (cigars per day), inhalation (any or the reported level), and duration of cigar smoking.

### All-cause mortality

All-cause mortality results are presented in Tables 2 and 3. Two studies reported all-cause mortality risk among primary cigar smokers. Shanks and Burns found a significant positive association (MR = 1.08, 95% CI = 1.05-1.12) based on 3,754 cigar smoker deaths in CPS-I, whereas Ben-Shlomo reported an inverse association between cigar smoking and all-cause mortality based on a much smaller cohort with only 9 cigar smoker deaths (Table 2). In three cigar studies that also included pipe smoking, primary pipe/cigar smoking was associated with slight increases in all-cause mortality risks (MRs 1.09 to 1.37), and all of these associations just missed conventional thresholds for statistical significance with the lower bounds of the 95% confidence intervals being at least 0.98. All-cause mortality risks were increased in two studies reporting risks for secondary cigar smoking (MRs 1.12 to 1.20), and one association was statistically significant while the other just missed statistical significance with 0.99 as the lower bound of the 95% confidence interval. All three studies on secondary cigar/pipe smoking found significant increases in all-cause mortality (MRs 1.13 to 1.46). Overall, MRs for all-cause mortality among current cigar smokers ranged from 0.48 to 1.60 and 0.70 to 1.68 among current cigar/pipe smokers. Among primary cigar smokers in CPS-1, smoking 3 or more cigars per day (MRs 1.08-1.17) and higher levels of cigar smoke inhalation (MRs 1.19-1.60) were both associated with significantly increased risk of all-cause mortality (Table 3).

**Table 2:** **Current cigar smoking and all-cause mortality**

**Study**	**Cohort name**	**Sex**	**Cigar smoker deaths**	**Effect estimate**	**95% CI**	**Measure**	Primary/secondary*	**Adjustment**
***Current Cigar***								
**Best1966**	Canadian Study of Smoking and Health		196	1.06	(0.92, 1.22)	SMR		Age
**Kahn1966**	Dorn study		1532	1.1	(1.05, 1.16)	SMR		Age
**Cole1974**			33	1.15	(0.70, 1.90)	IRR		
**Carstensen1987**	Swedish Census Cohort		131	1.39	(1.16, 1.65)	SMR		Age, residence
**Lange1992**	Copenhagen City Heart Study	Men	326	1.6	(1.30, 2.00)	HR		Age
**Lange1992**	Copenhagen City Heart Study	Women	185	1.8	(1.40, 2.20)	HR		Age
**Ben-Shlomo1994**	Whitehall study		9	0.48	(0.25, 0.93)	IRR	Primary	Age
**Ben-Shlomo1994**	Whitehall study		132	1.2	(0.99, 1.46)	IRR	Secondary	Age
**Shanks1998**	CPS-I		3754	1.08	(1.05, 1.12)	IRR	Primary	Age
**Shanks1998**	CPS-I		1462	1.12	(1.06, 1.18)	IRR	Secondary	Age
***Current Cigar and/or Pipe***								
**Cole1974**			35	1.68	(1.03, 2.75)	IRR		
**Gordon1974**	Framingham		52	0.76	(0.51, 1.12)	IRR		
**Doll1976**	Male British Doctors		388	1.09	(0.98, 1.20)	SMR	Primary	Age
**Doll1976**	Male British Doctors		595	1.13†	(1.04, 1.22)	SMR	Secondary	Age
**Sandler1989**	Washington, County, MD	Men	504	1.2	(1.07, 1.35)	IRR		Age, marital status, education, quality of housing
**Sandler1989**	Washington, County, MD	Women	2	0.7	(0.18, 2.75)	IRR		Age, marital status, education, quality of housing
**Wald1997**	British United Provident Association		113	1.23	(0.99, 1.75)	HR	Primary	Age
**Wald1997**	British United Provident Association		69	1.33	(1.03, 1.73)	HR	Secondary	Age
**Shaper2003**	CPS-II		41	1.37	(0.99, 1.91)	HR	Primary	Age, BMI, blood pressure/hypertension, serum cholesterol
**Shaper2003**	CPS-II		145	1.46	(1.18, 1.79)	HR	Secondary	Age, BMI, blood pressure/hypertension, serum cholesterol

*Primary cigar smoking: current, exclusive cigar smoking with no previous history of cigarette or pipe smoking; primary cigar and/or pipe smoking: current, exclusive cigar and/or pipe smoking with no previous history of cigarette smoking; secondary cigar smoking: current, exclusive cigar smoking with previous history of cigarette or pipe smoking; secondary cigar and/or pipe smoking: current, exclusive cigar and/or pipe smoking with previous history of cigarette smoking.

†Based on corrected death rate of 1600 per 100,000 [43].

**Table 3:** **Current cigar smoking and all-cause mortality by inhalation level and cigars per day**

**Study**	**Cohort name**	**Inhalation level**	**Cigars per day**	**Effect estimate**	**95% CI**	**Measure**	Primary/secondary*
**Kahn1966**	Dorn study		<5	1.04	(0.98, 1.11)	SMR	
**Kahn1966**	Dorn study		5 to 8	1.17	(1.06, 1.29)	SMR	
**Kahn1966**	Dorn study		8+	1.49	(1.24, 1.77)	SMR	
**Shanks1998**	CPS-I		1 to 2	1.02	(0.97, 1.07)	IRR	Primary
**Shanks1998**	CPS-I		3 to 4	1.08	(1.02, 1.15)	IRR	Primary
**Shanks1998**	CPS-I		5+	1.17	(1.10, 1.24)	IRR	Primary
**Shanks1998**	CPS-I		1 to 2	1.02	(0.93, 1.12)	IRR	Secondary
**Shanks1998**	CPS-I		3 to 4	1.17	(1.07, 1.28)	IRR	Secondary
**Shanks1998**	CPS-I		5+	1.18	(1.08, 1.29)	IRR	Secondary
**Shanks1998**	CPS-I	None		1.04	(1.00, 1.08)	IRR	Primary
**Shanks1998**	CPS-I	Slight		1.19	(1.09, 1.30)	IRR	Primary
**Shanks1998**	CPS-I	Moderate-deep		1.6	(1.38, 1.84)	IRR	Primary
**Shanks1998**	CPS-I	None		1.04	(0.97, 1.11)	IRR	Secondary
**Shanks1998**	CPS-I	Slight		1.16	(1.04, 1.29)	IRR	Secondary
**Shanks1998**	CPS-I	Moderate-deep		1.33	(1.16, 1.51)	IRR	Secondary

*Primary cigar smoking: current, exclusive cigar smoking with no previous history of cigarette or pipe smoking; secondary cigar smoking: current, exclusive cigar smoking with previous history of cigarette or pipe smoking.

### Oral cancer

Results for mortality from oral cancer are presented in Tables 4 and 5. Shanks and Burns and Shapiro, Jacobs, and Thun both found significantlyelevated risk of

**Table 4:** **Current cigar smoking and oral cancer**

**Study**	**Cohort name**	**Cigar smoker deaths**	**Effect estimate**	**95% CI**	**Measure**	Primary/secondary*	**Adjustment**	**ICD codes**
***Current Cigar***								
**Kahn1966**	Dorn study	9	4.11	(1.86, 7.84)	SMR		Age	ICD 7: 140-150
**Shanks1998**	CPS-I	25	7.92	(5.12, 11.69)	IRR	Primary	Age	
**Shanks1998**	CPS-I	8	6.58	(2.83, 12.97)	IRR	Secondary	Age	
**Shapiro2000**	CPS-II	6	4	(1.50, 10.30)	HR	Primary	Age, alcohol, smokeless tobacco	ICD 9: 140–141, 143-149

*Primary cigar smoking: current, exclusive cigar smoking with no previous history of cigarette or pipe smoking; secondary cigar smoking: current, exclusive cigar smoking with previous history of cigarette or pipe smoking.

**Table 5:** **Current cigar smoking and oral cancer by inhalation level, cigars per day, and duration**

**Study**	**Cohort name**	**Inhalation level**	**Cigars per day**	**Duration (years)**	**Effect estimate**	**95% CI**	**Measure**	Primary/secondary*
**Shanks1998**	CPS-I		1 to 2		2.12	(0.43, 6.18)	IRR	Primary
**Shanks1998**	CPS-I		3 to 4		8.51	(3.66, 16.77)	IRR	Primary
**Shanks1998**	CPS-I		5+		15.94	(8.71, 26.75)	IRR	Primary
**Shanks1998**	CPS-I		1 to 2		4.39	(0.06, 24.45)	IRR	Secondary
**Shanks1998**	CPS-I		5+		13.73	(5.50, 28.30)	IRR	Secondary
**Shapiro2000**	CPS-II		1 to 2		0	(0.00, 0.00)	HR	Primary
**Shapiro2000**	CPS-II		3+		7.6	(2.90, 19.60)	HR	Primary
**Shanks1998**	CPS-I	None			6.98	(4.13, 11.03)	IRR	Primary
**Shanks1998**	CPS-I	Slight			7.83	(1.57, 22.88)	IRR	Primary
**Shanks1998**	CPS-I	Moderate-deep			27.88	(5.60, 81.46)	IRR	Primary
**Shanks1998**	CPS-I	None			3.27	(0.66, 9.56)	IRR	Secondary
**Shanks1998**	CPS-I	Slight			8.75	(1.76, 25.58)	IRR	Secondary
**Shanks1998**	CPS-I	Moderate-deep			24.19	(2.72, 87.32)	IRR	Secondary
**Shapiro2000**	CPS-II	No inhalation			3.2	(0.90, 11.00)	HR	Primary
**Shapiro2000**	CPS-II	Inhalation			6.5	(1.40, 29.20)	HR	Primary
**Shapiro2000**	CPS-II			<25	0	(0.00, 0.00)	HR	Primary
**Shapiro2000**	CPS-II			25+	4.6	(1.60, 13.00)	HR	Primary

*Primary cigar smoking: current, exclusive cigar smoking with no previous history of cigarette or pipe smoking; secondary cigar smoking: current, exclusive cigar smoking with previous history of cigarette or pipe smoking.

oral cancer mortality for primary cigar smokers in CPS-I and II (Table 4). Secondary cigar smoking was also significantly associated with death from oral cancer in CPS-I. Overall, current cigar smoking MRs ranged from 4 to 7.9. Strong dose–response trends were observed for cigars per day, level of inhalation, and duration in both studies of primary cigar smoking in CPS-I and II (Table 5). Primary cigar smokers reporting no inhalation also had significantly elevated risk of oral cancer mortality in CPS-I (MR = 6.98, 95% CI = 4.13-11.03). Risk of oral cancer was elevated but not statistically significant (MR = 2.12, 95% CI = 0.43-6.18) among primary cigar smokers who smoked 1–2 cigars per day in CPS-I, presumably due to small sample size, based on the wide confidence interval. Risk could not be estimated in CPS-II due to 0 exposed cases smoking 1–2 cigars per day.

### Esophageal cancer

Results for deaths from esophageal cancer are presented in Tables 6 and 7. Shanks and Burns observed a significant positive association between primary cigar smoking and esophageal cancer mortality in CPS-I (MR = 3.60, 95% CI = 2.17-5.62) based on 19 exposed cases (Table 6). Shapiro, Jacobs, and Thun observed an elevated, but non-significant association in CPS-II (MR = 1.80, 95% CI = 0.90-3.70) based on 9 exposed cases, and was thus underpowered to detect this association. Shanks and Burns also observed a significant association for secondary cigar smoking in CPS-I. Current cigar smoking MRs ranged from 1.8 to 6.5. In CPS-I, Shanks and Burns found strong dose–response trends for cigars per day and depth of inhalation among primary cigar smokers (Table 7), even though an elevated risk was still observed among those reporting no inhalation (MR = 3.40, 95% CI = 1.90, 5.61). Risk of esophageal cancer was elevated but not statistically significant among primary cigar smokers who smoked 1–2 cigars per day in both CPS-I (MR = 2.28, 95% CI = 0.74-5.33) and CPS-II (MR = 1.80, 95% CI = 0.60-5.00, based on 4 exposed cases). Based on the wide confidence intervals, both cohorts appear to be underpowered to detect an association at this level of exposure due to small sample size.

**Table 6:** **Current cigar smoking and esophageal cancer**

**Study**	**Cohort name**	**Cigar smoker deaths**	**Effect estimate**	**95% CI**	**Measure**	Primary/secondary*	**Adjustment**	**ICD codes**
***Current Cigar***								
**Kahn1966**	Dorn study	12	5.33	(2.74, 9.34)	SMR		Age	ICD 8: 150
**Carstensen1987**	Swedish Census Cohort	2	6.5	(0.61, 23.90)	SMR		Age, residence	ICD 8: 150
**Shanks1998**	CPS-I	19	3.6	(2.17, 5.62)	IRR	Primary	Age	
**Shanks1998**	CPS-I	7	3.52	(1.41, 7.25)	IRR	Secondary	Age	
**Shapiro2000**	CPS-II	9	1.8	(0.90, 3.70)	HR	Primary	Age, alcohol, smokeless tobacco	ICD 9: 150

*Primary cigar smoking: current, exclusive cigar smoking with no previous history of cigarette or pipe smoking; secondary cigar smoking: current, exclusive cigar smoking with previous history of cigarette or pipe smoking.

**Table 7:** **Current cigar smoking and esophageal cancer by inhalation level, cigars per day, and duration**

**Study**	**Cohort name**	**Inhalation level**	**Cigars per day**	**Duration (years)**	**Effect estimate**	**95% CI**	**Measure**	Primary/secondary*
**Shanks1998**	CPS-I		1 to 2		2.28	(0.74, 5.33)	IRR	Primary
**Shanks1998**	CPS-I		3 to 4		3.93	(1.43, 8.55)	IRR	Primary
**Shanks1998**	CPS-I		5+		5.19	(2.23, 10.22)	IRR	Primary
**Shanks1998**	CPS-I		1 to 2		2.64	(0.03, 14.67)	IRR	Secondary
**Shanks1998**	CPS-I		3 to 4		1.56	(0.02, 8.68)	IRR	Secondary
**Shanks1998**	CPS-I		5+		5.63	(1.81, 13.14)	IRR	Secondary
**Shapiro2000**	CPS-II		1 to 2		1.8	(0.60, 5.00)	HR	Primary
**Shapiro2000**	CPS-II		3+		1.9	(0.80, 4.90)	HR	Primary
**Shanks1998**	CPS-I	None			3.4	(1.90, 5.61)	IRR	Primary
**Shanks1998**	CPS-I	Slight			1.9	(0.02, 10.58)	IRR	Primary
**Shanks1998**	CPS-I	Moderate-deep			14.84	(2.98, 43.37)	IRR	Primary
**Shanks1998**	CPS-I	None			4.15	(1.34, 9.68)	IRR	Secondary
**Shanks1998**	CPS-I	Slight			2.22	(0.03, 12.37)	IRR	Secondary
**Shanks1998**	CPS-I	Moderate-deep			2.69	(0.04, 14.94)	IRR	Secondary
**Shapiro2000**	CPS-II	No inhalation			1.6	(0.70, 4.10)	HR	Primary
**Shapiro2000**	CPS-II	Inhalation			1	(0.10, 7.20)	HR	Primary
**Shapiro2000**	CPS-II			<25	0.9	(0.10, 6.40)	HR	Primary
**Shapiro2000**	CPS-II			25+	2.2	(1.00, 4.70)	HR	Primary

*Primary cigar smoking: current, exclusive cigar smoking with no previous history of cigarette or pipe smoking; secondary cigar smoking: current, exclusive cigar smoking with previous history of cigarette or pipe smoking.

### Stomach cancer

Mortality risk estimates for stomach cancer are presented in Tables 8 and 9. Overall, current cigar smoking MRs were 1.2 in the Dorn study cohort and 2.29 from CPS-II, with the estimate from CPS-II being statistically significant (Table 8). Stronger associations were observed for the highest levels of both cigars per day and inhalation in CPS-II (Table 9). Cigar smoking was found to be associated with stomach cancer mortality in CPS-II for both less than 40 years of cigar smoking and 40 years or more of cigar smoking.

**Table 8:** **Current cigar smoking and stomach cancer**

**Study**	**Cohort name**	**Cigar smoker deaths**	**Effect estimate**	**95% CI**	**Measure**	**Adjustment**	**ICD codes**
***Current Cigar***							
**Kahn1966**	Dorn Study	23	1.2	(0.76, 1.80)	SMR	Age	ICD 7: 151
**Chao2002**	CPS-II	25	2.29	(1.49, 3.51)	HR	Age, education, race, family history of stomach cancer,high-fiber grain foods, vegetables, citrus fruits/juices,	ICD 9: 151.0-151.9

**Table 9:** **Current cigar smoking and stomach cancer by inhalation level, cigars per day, duration, and age started smoking cigars**

**Study**	**Cohort name**	**Inhalation level**	**Cigars per day**	**Duration (years)**	**Age started cigars (years)**	**Effect estimate**	**95% CI**	**P-trend**	**Measure**
**Chao2002**	CPS-II		1 to 4			1.68	(0.95, 2.97)		HR
**Chao2002**	CPS-II		5+			4.2	(2.32, 7.60)	0.746	HR
**Chao2002**	CPS-II	None				2.08	(1.22, 3.57)		HR
**Chao2002**	CPS-II	Slight to deep				3.93	(1.92, 8.04)		HR
**Chao2002**	CPS-II			≤39		2.42	(1.36, 4.28)		HR
**Chao2002**	CPS-II			≥40		2.55	(1.32, 4.94)	0.079	HR
**Chao2002**	CPS-II				≤19	2.78	1.23 6.28		HR
**Chao2002**	CPS-II				20-29	3.01	1.65 5.49		HR
**Chao2002**	CPS-II				≥30	1.74	0.71 4.24	0.617	HR

### Liver cancer

Results for liver cancer are presented in Table 10. Studies of liver cancer mortality risk for cigar smoking are limited. Both studies of current cigar smoking showed significant associations with deaths from liver cancer; however, neither study reported cigarette smoking history.

**Table 10:** **Current cigar smoking and liver cancer**

**Study**	**Cohort name**	**Cigar smoker deaths**	**Effect estimate**	**95% CI**	**Measure**	**Adjustment**	**ICD codes**
**Carstensen1987**	Swedish Census Cohort	4	7.2	(1.87, 18.62)	SMR	Age, residence	ICD 8: 155-156
**Hsing1990**	Dorn study	47	3.1	(2.00, 4.80)	IRR	Age, calendar time	ICD 7: 155.0

### Pancreatic cancer

Results for deaths from pancreatic cancer are presented in Tables 11 and 12. Shanks and Burns found a significant association between primary cigar smoking and pancreatic cancer mortality in CPS-I, while Shapiro, Jacobs, and Thun did not find a significant association in CPS-II (Table 11). Shanks and Burns also found a significant association with pancreatic cancer mortality risk for secondary cigar smokers. Overall, current cigar smoking MRs ranged from 1 to 1.8. A weak dose–response trend was seen for cigars per day in CPS-I and II (Table 12). Inhalation was associated with pancreatic cancer mortality in CPS-II, and some evidence of a dose–response trend for inhalation was observed in CPS-I.

**Table 11:** **Current cigar smoking and pancreatic cancer**

**Study**	**Cohort name**	**Cigar smoker deaths**	**Effect estimate**	**95% CI**	**Measure**	Primary/secondary*	**Adjustment**	**ICD codes**
***Current Cigar***								
**Kahn1966**	Dorn study	27	1.52	(1.00, 2.21)	SMR		Age	ICD 7: 157
**Carstensen1987**	Swedish Census Cohort	1	1	(0.00, 5.73)	SMR		Age, residence	ICD 8: 140–146, 148, 161
**Shanks1998**	CPS-I	56	1.62	(1.22, 2.11)	IRR	Primary	Age	
**Shanks1998**	CPS-I	20	1.8	(1.10, 2.78)	IRR	Secondary	Age	
**Shapiro2000**	CPS-II	28	1.3	(0.90, 1.90)	HR	Primary	Age, alcohol, smokeless tobacco	ICD 9: 157

*Primary cigar smoking: current, exclusive cigar smoking with no previous history of cigarette or pipe smoking; secondary cigar smoking: current, exclusive cigar smoking with previous history of cigarette or pipe smoking.

**Table 12:** **Current cigar smoking and pancreatic cancer by inhalation level, cigars per day, and duration**

**Study**	**Cohort name**	**Inhalation level**	**Cigars per day**	**Duration (years)**	**Effect estimate**	**95% CI**	**Measure**	Primary/secondary*
**Shanks1998**	CPS-I		1 to 2		1.18	(0.69, 1.89)	IRR	Primary
**Shanks1998**	CPS-I		3 to 4		1.51	(0.86, 2.45)	IRR	Primary
**Shanks1998**	CPS-I		5+		2.21	(1.40, 3.32)	IRR	Primary
**Shanks1998**	CPS-I		1 to 2		0.56	(0.06, 2.01)	IRR	Secondary
**Shanks1998**	CPS-I		3 to 4		1.9	(0.82, 3.74)	IRR	Secondary
**Shanks1998**	CPS-I		5+		3.71	(1.78, 6.83)	IRR	Secondary
**Shapiro2000**	CPS-II		1 to 2		0.6	(0.30, 1.40)	HR	Primary
**Shapiro2000**	CPS-II		3+		1.6	(1.00, 2.50)	HR	Primary
**Shanks1998**	CPS-I	None			1.55	(1.12, 2.07)	IRR	Primary
**Shanks1998**	CPS-I	Slight			2.16	(0.99, 4.10)	IRR	Primary
**Shanks1998**	CPS-I	Moderate-deep			2.26	(0.45, 6.60)	IRR	Primary
**Shanks1998**	CPS-I	None			1.55	(0.80, 2.72)	IRR	Secondary
**Shanks1998**	CPS-I	Slight			1.92	(0.52, 4.92)	IRR	Secondary
**Shanks1998**	CPS-I	Moderate-deep			2.53	(0.51, 7.39)	IRR	Secondary
**Shapiro2000**	CPS-II	No inhalation			0.9	(0.50, 1.50)	HR	Primary
**Shapiro2000**	CPS-II	Inhalation			2.7	(1.50, 4.80)	HR	Primary
**Shapiro2000**	CPS-II			<25	1.5	(0.70, 3.30)	HR	Primary
**Shapiro2000**	CPS-II			25+	1.1	(0.70, 1.80)	HR	Primary

*Primary cigar smoking: current, exclusive cigar smoking with no previous history of cigarette or pipe smoking; secondary cigar smoking: current, exclusive cigar smoking with previous history of cigarette or pipe smoking.

### Laryngeal cancer

The results for deaths from laryngeal cancer are presented in Tables 13 and 14. Shanks and Burns and Shapiro, Jacobs, and Thun found significant positive associations between primary cigar smoking and laryngeal cancer mortality risk in CPS-I and CPS-II that were of similar magnitude (MRs = 10) (Table 13). Kahn also found a significant association of the same magnitude among current cigar smokers. Shanks and Burns and Shapiro, Jacobs, and Thun observed a strong dose–response relationship for cigars per day and level of inhalation, and Shapiro, Jacobs, and Thun observed similar trends for duration of cigar smoking (Table 14). Shanks and Burns also observed that primary cigar smokers reporting no inhalation in CPS-I had significantly elevated risk of mortality from laryngeal cancer (MR = 10.6, 95% CI = 3.87-23.07). Risk of laryngeal cancer was highly elevated but not statistically significant among primary cigar smokers who smoked 1–2 cigars per day in both CPS-I (MR = 6.45, 95% CI = 0.72-23.27) and CPS-II (MR = 6.00, 95% CI-0.70-53.50, based on 1 exposed case). Based on the wide confidence intervals, both cohorts appear to be underpowered to detect an association at this level of exposure due to small sample size.

**Table 13:** **Current cigar smoking and laryngeal cancer**

**Study**	**Cohort name**	**Cigar smoker deaths**	**Effect estimate**	**95% CI**	**Measure**	Primary/secondary*	**Adjustment**	**ICD codes**
***Current Cigar***								
**Kahn1966**	Dorn study	6	10.33	(3.72, 22.63)	SMR		Age	ICD 7: 161
**Shanks1998**	CPS-I	7	10.02	(4.01, 20.64)	IRR	Primary	Age	
**Shapiro2000**	CPS-II	4	10.3	(2.60, 41.00)	HR	Primary	Age, alcohol, smokeless tobacco	ICD 9: 161

*Primary cigar smoking: current, exclusive cigar smoking with no previous history of cigarette or pipe smoking; secondary cigar smoking: current, exclusive cigar smoking with previous history of cigarette or pipe smoking.

**Table 14:** **Current cigar smoking and laryngeal cancer by inhalation level, cigars per day, and duration**

**Study**	**Cohort name**	**Inhalation level**	**Cigars per day**	**Duration (years)**	**Effect estimate**	**95% CI**	**Measure**	Primary/secondary*
**Shanks1998**	CPS-I		1 to 2		6.45	(0.72, 23.27)	IRR	Primary
**Shanks1998**	CPS-I		5+		26.03	(8.39, 60.74)	IRR	Primary
**Shapiro2000**	CPS-II		1 to 2		6	(0.70, 53.50)	HR	Primary
**Shapiro2000**	CPS-II		3+		15	(3.40, 65.90)	HR	Primary
**Shanks1998**	CPS-I	None			10.6	(3.87, 23.07)	IRR	Primary
**Shanks1998**	CPS-I	Moderate-deep			53.26	(0.70, 296.32)	IRR	Primary
**Shapiro2000**	CPS-II	No inhalation			4.2	(0.50, 37.10)	HR	Primary
**Shapiro2000**	CPS-II	Inhalation			39	(8.40, 180.10)	HR	Primary
**Shapiro2000**	CPS-II			<25	0	(0.00, 0.00)	HR	Primary
**Shapiro2000**	CPS-II			25+	13.7	(3.40, 54.50)	HR	Primary

*Primary cigar smoking: current, exclusive cigar smoking with no previous history of cigarette or pipe smoking; secondary cigar smoking: current, exclusive cigar smoking with previous history of cigarette or pipe smoking.

### Lung cancer

Lung cancer mortality risk estimates are presented in Tables 15 and 16. Shanks and Burns and Shapiro, Jacobs, and Thun found significant positive associations with lung cancer mortality risk in CPS-I and II, although Ben-Shlomo did not observe a significant association in a much smaller cohort (Table 15). Wald found a significant association between primary cigar/pipe smoking and lung cancer mortality risk. Shanks and Burns and Ben-Shlomo found a significant positive association between lung cancer mortality and secondary cigar smoking as did Wald for secondary cigar/pipe smoking. Overall, current cigar smoking MRs ranged from 1.59 to 7.64, and current cigar/pipe smoking MRs ranged from 3.19 to 8.64. Dose–response trends were observed for cigars per day, level of inhalation, and duration in CPS-I and II data (Table 16).

**Table 15:** **Current cigar smoking and lung cancer**

**Study**	**Cohort name**	**Sex**	**Cigar smoker deaths**	**Effect estimate**	**95% CI**	**Measure**	Primary/secondary*	**Adjustment**	**ICD codes**
***Current Cigar***									
**Kahn1966**	**Dorn study**		25	1.59	(1.03, 2.35)	SMR		Age	ICD 7: 162-163
**Carstensen1987**	**Swedish Census Cohort**		11	7.6	(3.77, 13.65)	SMR		Age, residence	ICD 8: 162
**Lange1992**	**Copenhagen City Heart Study**	Men	47	6	(2.20, 17.00)	HR		Age	ICD 8: 162
**Lange1992**	**Copenhagen City Heart Study**	Women	14	4.9	(3.00,12.00)	HR		Age	ICD 8: 162
**Ben-Shlomo1994**	**Whitehall study**		1	1.8	(0.24, 13.31)	IRR	Primary	Age	ICD 8: 162
**Ben-Shlomo1994**	**Whitehall study**		20	7.64	(4.22, 13.83)	IRR	Secondary	Age	ICD 8: 162
**Shanks1998**	**CPS-I**		73	2.1	(1.63, 2.65)	IRR	Primary	Age	
**Shanks1998**	**CPS-I**		83	6.29	(5.01, 7.79)	IRR	Secondary	Age	
**Shapiro2000**	**CPS-II**		88	5.1	(4.00, 6.60)	HR	Primary	Age, alcohol, smokeless tobacco	ICD 9: 162
***Current Cigar and/or Pipe***									
**Chow1992**	**Dorn study**		4	3.5	(1.00, 12.60)	HR		Age, occupation	
**Wald1997**	**British United Provident Association**		6	3.19	(1.07, 9.50)	HR	Primary	Age	ICD 9: 162
**Wald1997**	**British United Provident Association**		9	8.64	(3.19, 23.30)	HR	Secondary	Age	ICD 9: 162

*Primary cigar smoking: current, exclusive cigar smoking with no previous history of cigarette or pipe smoking; primary cigar and/or pipe smoking: current, exclusive cigar and/or pipe smoking with no previous history of cigarette smoking; secondary cigar smoking: current, exclusive cigar smoking with previous history of cigarette or pipe smoking; secondary cigar and/or pipe smoking: current, exclusive cigar and/or pipe smoking with previous history of cigarette smoking.

**Table 16:** **Current cigar smoking and lung cancer by inhalation level, cigars per day, and duration**

**Study**	**Cohort name**	**Inhalation level**	**Cigars per day**	**Duration (years)**	**Effect estimate**	**95% CI**	**Measure**	Primary/secondary*
**Kahn1966**	Dorn study		<5		1.14	(0.59, 2.00)	SMR	
**Kahn1966**	Dorn study		5 to 8		2.64	(1.31, 4.74)	SMR	
**Kahn1966**	Dorn study		8+		2.07	(0.20, 7.61)	SMR	
**Shanks1998**	CPS-I		1 to 2		0.9	(0.54, 1.66)	IRR	Primary
**Shanks1998**	CPS-I		3 to 4		2.36	(1.49, 3.54)	IRR	Primary
**Shanks1998**	CPS-I		5+		3.4	(2.34, 4.77)	IRR	Primary
**Shanks1998**	CPS-I		1 to 2		3.18	(1.78, 5.24)	IRR	Secondary
**Shanks1998**	CPS-I		3 to 4		8.52	(5.87, 11.97)	IRR	Secondary
**Shanks1998**	CPS-I		5+		7.21	(5.02, 10.03)	IRR	Secondary
**Shapiro2000**	CPS-II		1 to 2		1.3	(0.70, 2.40)	HR	Primary
**Shapiro2000**	CPS-II		3+		7.8	(5.90, 10.30)	HR	Primary
**Shanks1998**	CPS-I	None			1.97	(1.48, 2.57)	IRR	Primary
**Shanks1998**	CPS-I	Slight			1.89	(0.81, 3.72)	IRR	Primary
**Shanks1998**	CPS-I	Moderate-deep			4.93	(1.80, 10.72)	IRR	Primary
**Shanks1998**	CPS-I	None			5.41	(3.93, 7.27)	IRR	Secondary
**Shanks1998**	CPS-I	Slight			7.63	(4.66, 11.78)	IRR	Secondary
**Shanks1998**	CPS-I	Moderate-deep			9.77	(5.88, 15.25)	IRR	Secondary
**Shapiro2000**	CPS-II	No inhalation			3.3	(2.30, 4.70)	HR	Primary
**Shapiro2000**	CPS-II	Inhalation			11.3	(7.90, 16.10)	HR	Primary
**Shapiro2000**	CPS-II			<25	2.1	(1.00, 4.20)	HR	Primary
**Shapiro2000**	CPS-II			25+	5.9	(4.50, 7.70)	HR	Primary

*Primary cigar smoking: current, exclusive cigar smoking with no previous history of cigarette or pipe smoking; secondary cigar smoking: current, exclusive cigar smoking with previous history of cigarette or pipe smoking.

### Bladder cancer

The results for deaths from bladder cancer are presented in Tables 17 and 18. Neither Shanks and Burns nor Shapiro, Jacob, and Thun found an association between primary cigar smoking and bladder cancer mortality risk in CPS-I and CPS-II, respectively, and Shanks and Burns did not find an association with secondary cigar smoking (Table 17). Overall, current cigar smoking MRs ranged from 0.94 to 1.9. There was no dose–response trend for cigars per day among primary and secondary cigar smokers in both CPS-I and CPS-II. Shanks and Burns did not find increased mortality risk with depth of inhalation in CPS-I, but Shapiro, Jacobs, and Thun did find a significant association with inhalation among primary cigar smokers (Table 18).

**Table 17:** **Current cigar smoking and bladder cancer**

**Study**	**Cohort name**	**Cigar smoker deaths**	**Effect estimate**	**95% CI**	**Measure**	Primary/secondary*	**Adjustment**	**ICD codes**
***Current Cigar***								
**Kahn1966**	Dorn study	10	0.94	(0.45, 1.74)	SMR		Age	ICD 7: 181
**Carstensen1987**	Swedish Census Cohort	1	1.9	(0.00, 10.89)	SMR		Age, residence	ICD 8: 188
**Shanks1998**	CPS-I	25	1.38	(0.89, 2.04)	IRR	Primary	Age	
**Shanks1998**	CPS-I	9	1.23	(0.56, 2.33)	IRR	Secondary	Age	
**Shapiro2000**	CPS-II	6	1	(0.40, 2.30)	HR	Primary	Age, alcohol, smokeless tobacco	ICD 9: 188

*Primary cigar smoking: current, exclusive cigar smoking with no previous history of cigarette or pipe smoking; secondary cigar smoking: current, exclusive cigar smoking with previous history of cigarette or pipe smoking.

**Table 18:** **Current cigar smoking and bladder cancer by inhalation level, cigars per day, and duration**

**Study**	**Cohort name**	**Inhalation level**	**Cigars per day**	**Duration (years)**	**Effect estimate**	**95% CI**	**Measure**	Primary/secondary*
**Shanks1998**	CPS-I		1 to 2		0.78	(0.29, 1.71)	IRR	Primary
**Shanks1998**	CPS-I		3 to 4		1.68	(0.77, 3.18)	IRR	Primary
**Shanks1998**	CPS-I		5+		2.03	(0.97, 3.73)	IRR	Primary
**Shanks1998**	CPS-I		1 to 2		1.02	(0.20, 2.97)	IRR	Secondary
**Shanks1998**	CPS-I		3 to 4		2.36	(0.76, 5.50)	IRR	Secondary
**Shanks1998**	CPS-I		5+		0.32	(0.00, 1.80)	IRR	Secondary
**Shapiro2000**	CPS-II		1 to 2		0	(0.00, 0.00)	HR	Primary
**Shapiro2000**	CPS-II		3+		1.9	(0.80, 4.40)	HR	Primary
**Shanks1998**	CPS-I	None			1.57	(1.00, 2.36)	IRR	Primary
**Shanks1998**	CPS-I	Moderate-deep			1.52	(0.02, 8.44)	IRR	Primary
**Shanks1998**	CPS-I	None			0.77	(0.21, 1.98)	IRR	Secondary
**Shanks1998**	CPS-I	Slight			2.87	(0.58, 8.40)	IRR	Secondary
**Shanks1998**	CPS-I	Moderate-deep			1.45	(0.16, 5.25)	IRR	Secondary
**Shapiro2000**	CPS-II	No inhalation			0.5	(0.10, 2.10)	HR	Primary
**Shapiro2000**	CPS-II	Inhalation			3.6	(1.30, 9.90)	HR	Primary
**Shapiro2000**	CPS-II			<25	0	(0.00, 0.00)	HR	Primary
**Shapiro2000**	CPS-II			25+	1.1	(0.40, 2.70)	HR	Primary

*Primary cigar smoking: current, exclusive cigar smoking with no previous history of cigarette or pipe smoking; secondary cigar smoking: current, exclusive cigar smoking with previous history of cigarette or pipe smoking.

### Coronary heart disease

Mortality results due to coronary heart disease (CHD) are presented in Tables 19 and 20. Primary cigar smoking was significantly associated with CHD mortality in CPS-I (MR = 1.05, 95% CI = 1.00-1.11). Primary cigar smoking was also associated with CHD mortality in CPS-II men aged 30–74 years (MR = 1.30, 95% CI = 1.05-1.62), but not in men 75 years and older. Primary cigar smoking was also not associated with CHD mortality in the much smaller Whitehall study cohort. The one study on primary cigar/pipe smoking from Wald did not observe an increased risk of death from CHD. CPS-I also found increased CHD mortality risk to be associated with secondary cigar smoking (MR = 1.09, 95% CI = 1.01-1.18), although the small Whitehall study cohort did not. Wald did not find an association between secondary cigar/pipe smoking and CHD mortality risk. Overall, the current cigar smoking MRs ranged from 0.45 to 1.30, and the current cigar/pipe MRs ranged from 0.98 to 1.67. There was a weak dose–response relationship with cigars per day and level of inhalation among primary but not secondary cigar smokers in the CPS-I study (Table 20). Primary cigar smoking for 25 years or more was also associated with increased CHD mortality risk in CPS-II men aged 30–74 years.

**Table 19:** **Current cigar smoking and coronary heart disease**

**Study**	**Cohort name**	**Cigar smoker deaths**	**Effect estimate**	**95% CI**	**Measure**	Primary/secondary*	**Adjustment**	**ICD codes**
***Current Cigar***								
**Kahn1966**	Dorn study	623	1.04	(0.96, 1.13)	SMR		Age	ICD 7: 420
**Carstensen1987**	Swedish Census Cohort	42	1.16	(0.84, 1.57)	SMR		Age, residence	ICD 8: 410-414
**Ben-Shlomo1994**	Whitehall study	4	0.45	(0.17, 1.22)	IRR	Primary	Age	ICD 8: 410-414
**Ben-Shlomo1994**	Whitehall study	42	0.91	(0.65, 1.27)	IRR	Secondary	Age	ICD 8: 410-414
**Shanks1998**	CPS-I	1505	1.05	(1.00, 1.11)	IRR	Primary	Age	
**Shanks1998**	CPS-I	609	1.09	(1.01, 1.18)	IRR	Secondary	Age	
**Jacobs1999**	CPS-II (age 30–74 years)	98	1.30	(1.05, 1.62)	HR	Primary	Age, BMI, blood pressure/hypertension, alcohol consumption, education, exercise level, environmental tob smoke, vit C supplements	ICD 9: 410-414
**Jacobs1999**	CPS-II (age 75+ years)	64	0.93	(0.72, 1.21)	HR	Primary	Age, BMI, blood pressure/hypertension, alcohol consumption, education, exercise level, environmental tob smoke, vit C supplements	ICD 9: 410-414
***Current Cigar and/or Pipe***								
**Jajich1984**	Cook County, IL	32	1.67	(1.11, 2.50)	IRR			
**Wald1997**	British United Provident Association	33	0.98	(0.67, 1.44)	HR	Primary	Age	ICD 9: 410-414
**Wald1997**	British United Provident Association	25	1.29	(0.88, 1.99)	HR	Secondary	Age	ICD 9: 410-414

*Primary cigar smoking: current, exclusive cigar smoking with no previous history of cigarette or pipe smoking; primary cigar and/or pipe smoking: current, exclusive cigar and/or pipe smoking with no previous history of cigarette smoking; secondary cigar smoking: current, exclusive cigar smoking with previous history of cigarette or pipe smoking; secondary cigar and/or pipe smoking: current, exclusive cigar and/or pipe smoking with previous history of cigarette smoking.

**Table 20:** **Current cigar smoking and coronary heart disease by inhalation level, cigars per day and duration**

**Study**	**Cohort name**	**Inhalation level**	**Cigars per day**	**Duration (years)**	**Effect estimate**	**95% CI**	**Measure**	Primary/secondary*
**Kahn1966**	Dorn study		<5		1	(0.90, 1.10)	SMR	
**Kahn1966**	Dorn study		5 to 8		1.1	(0.94, 1.28)	SMR	
**Kahn1966**	Dorn study		8+		1.18	(0.86, 1.59)	SMR	
**Shanks1998**	CPS-I		1 to 2		0.98	(0.91, 1.07)	IRR	Primary
**Shanks1998**	CPS-I		3 to 4		1.06	(0.96, 1.16)	IRR	Primary
**Shanks1998**	CPS-I		5+		1.14	(1.03, 1.24)	IRR	Primary
**Shanks1998**	CPS-I		1 to 2		1.06	(0.92, 1.21)	IRR	Secondary
**Shanks1998**	CPS-I		3 to 4		1.1	(0.95, 1.27)	IRR	Secondary
**Shanks1998**	CPS-I		5+		1.1	(0.96, 1.26)	IRR	Secondary
**Jacobs1999**	CPS-II (age 30–74 years)		1		1.18	(0.76, 1.82)	HR	Primary
**Jacobs1999**	CPS-II (age 30–74 years)		2 to 3		1.43	(1.03, 1.99)	HR	Primary
**Jacobs1999**	CPS-II (age 30–74 years)		4+		1.33	(0.95, 1.86)	HR	Primary
**Jacobs1999**	CPS-II (age 75+ years)		1		1.07	(0.64, 1.78)	HR	Primary
**Jacobs1999**	CPS-II (age 75+ years)		2 to 3		0.72	(0.45, 1.16)	HR	Primary
**Jacobs1999**	CPS-II (age 75+ years)		4+		1.03	(0.70, 1.51)	HR	Primary
**Shanks1998**	CPS-I	None			1.01	(0.96, 1.07)	IRR	Primary
**Shanks1998**	CPS-I	Slight			1.23	(1.07, 1.41)	IRR	Primary
**Shanks1998**	CPS-I	Moderate-deep			1.37	(1.07, 1.75)	IRR	Primary
**Shanks1998**	CPS-I	None			1.02	(0.92, 1.13)	IRR	Secondary
**Shanks1998**	CPS-I	Slight			1.1	(0.93, 1.30)	IRR	Secondary
**Shanks1998**	CPS-I	Moderate-deep			1.23	(0.99, 1.51)	IRR	Secondary
**Jacobs1999**	CPS-II (age 30–74 years)	No inhalation			1.25	(0.96, 1.62)	HR	Primary
**Jacobs1999**	CPS-II (age 30–74 years)	Inhalation			1.6	(1.06, 2.41)	HR	Primary
**Jacobs1999**	CPS-II (age 75+ years)	No inhalation			0.87	(0.62, 1.22)	HR	Primary
**Jacobs1999**	CPS-II (age 75+ years)	Inhalation			0.93	(0.49, 1.75)	HR	Primary
**Jacobs1999**	CPS-II (age 30–74 years)			<25	1.03	(0.64, 1.65)	HR	Primary
**Jacobs1999**	CPS-II (age 30–74 years)			25+	1.42	(1.11, 1.82)	HR	Primary
**Jacobs1999**	CPS-II (age 75+ years)			<25	0.75	(0.28, 2.00)	HR	Primary
**Jacobs1999**	CPS-II (age 75+ years)			25+	0.95	(0.70, 1.28)	HR	Primary

*Primary cigar smoking: current, exclusive cigar smoking with no previous history of cigarette or pipe smoking; secondary cigar smoking: current, exclusive cigar smoking with previous history of cigarette or pipe smoking.

### Stroke

Results for stroke are presented in Tables 21 and 22. Shanks and Burns did not find a significant association with stroke mortality risk for either primary or secondary cigar smoking in CPS-I (Table 21). Overall, current cigar smoking MRs ranged from 0.92 to 1.08, and Haheim found a significantly increased risk of death from stroke for cigar/pipe smoking (MR = 3.6, 95% CI = 1.05-12.3). However, there was no dose–response trend for cigars per day and level of inhalation among primary and secondary cigar smokers in CPS-I (Table 22).

**Table 21:** **Current cigar smoking and stroke**

**Study**	**Cohort name**	**Cigar smoker deaths**	**Effect estimate**	**95% CI**	**Measure**	Primary/secondary*	**Adjustment**	**ICD codes**
***Current Cigar***								
**Kahn1966**	Dorn study	135	1.08	(0.91, 1.28)	SMR		Age	ICD 7: 330-334
**Shanks1998**	CPS-I	431	0.96	(0.87, 1.06)	IRR	Primary	Age	
**Shanks1998**	CPS-I	133	0.92	(0.77, 1.09)	IRR	Secondary	Age	
***Current Cigar and/or Pipe***								
**Haheim1996**	Oslo study	7	3.6	(1.05, 12.30)	HR		Age, blood pressure/hypertension, fasting glucose/diabetes	ICD 8, 9: 430-438

*Primary cigar smoking: current, exclusive cigar smoking with no previous history of cigarette or pipe smoking; primary cigar and/or pipe smoking: current, exclusive cigar and/or pipe smoking with no previous history of cigarette smoking; secondary cigar smoking: current, exclusive cigar smoking with previous history of cigarette or pipe smoking; secondary cigar and/or pipe smoking: current, exclusive cigar and/or pipe smoking with previous history of cigarette smoking.

**Table 22:** **Current cigar smoking and stroke by inhalation level and cigars per day**

**Study**	**Cohort name**	**Inhalation level**	**Cigars per day**	**Effect estimate**	**95% CI**	**Measure**	Primary/secondary*
**Shanks1998**	CPS-I		1 to 2	1.01	(0.88, 1.17)	IRR	Primary
**Shanks1998**	CPS-I		3 to 4	1.05	(0.88, 1.23)	IRR	Primary
**Shanks1998**	CPS-I		5+	0.79	(0.64, 0.97)	IRR	Primary
**Shanks1998**	CPS-I		1 to 2	0.95	(0.71, 1.26)	IRR	Secondary
**Shanks1998**	CPS-I		3 to 4	0.92	(0.67, 1.24)	IRR	Secondary
**Shanks1998**	CPS-I		5+	0.89	(0.64, 1.22)	IRR	Secondary
**Shanks1998**	CPS-I	None		0.91	(0.82, 1.02)	IRR	Primary
**Shanks1998**	CPS-I	Slight		1.06	(0.79, 1.39)	IRR	Primary
**Shanks1998**	CPS-I	Moderate-deep		1.22	(0.74, 1.91)	IRR	Primary
**Shanks1998**	CPS-I	None		0.95	(0.76, 1.18)	IRR	Secondary
**Shanks1998**	CPS-I	Slight		0.83	(0.55, 1.20)	IRR	Secondary
**Shanks1998**	CPS-I	Moderate-deep		0.88	(0.52, 1.38)	IRR	Secondary

*Primary cigar smoking: current, exclusive cigar smoking with no previous history of cigarette or pipe smoking; secondary cigar smoking: current, exclusive cigar smoking with previous history of cigarette or pipe smoking.

### Aortic aneurysm

Mortality risk estimates for aortic aneurysm are presented in Tables 23 and 24. Shanks and Burns found significant positive associations for aortic aneurysm mortality for both primary and secondary cigar smoking in CPS-I (Table 23). Overall, current cigar smoking MRs ranged from 1.76 to 5.10, while Strachan found that current cigar/pipe smoking was associated with a five-fold increased risk of death from aortic aneurysm (MR = 5.40, 95% CI = 1.90-15.30). There were no clear dose–response trends for cigars per day and levels of inhalation with in CPS-I (Table 24). Notably, risk of aortic aneurysm was significantly elevated among primary cigar smokers who smoked 1–2 cigars per day in CPS-I (MR = 1.82, 95% CI = 1.11-2.81).

**Table 23:** **Current cigar smoking and aortic aneurysm**

**Study**	**Cohort name**	**Cigar smoker deaths**	**Effect estimate**	**95% CI**	**Measure**	Primary/secondary*	**Adjustment**	**ICD codes**
***Current Cigar***								
**Kahn1966**	Dorn study	24	2.06	(1.32, 3.07)	SMR		Age	ICD 7: 450
**Carstensen1987**	Swedish Census Cohort	4	5.1	(1.33, 13.19)	SMR		Age, residence	ICD 8: 451
**Shanks1998**	CPS-I	46	1.76	(1.29, 2.35)	IRR	Primary	Age	
**Shanks1998**	CPS-I	31	2.82	(1.91, 4.00)	IRR	Secondary	Age	
***Current Cigar and/or Pipe***								
**Strachan1991**	Whitehall study		5.4	(1.90, 15.30)	OR		Age	ICD 8: 441

*Primary cigar smoking: current, exclusive cigar smoking with no previous history of cigarette or pipe smoking; primary cigar and/or pipe smoking: current, exclusive cigar and/or pipe smoking with no previous history of cigarette smoking; secondary cigar smoking: current, exclusive cigar smoking with previous history of cigarette or pipe smoking; secondary cigar and/or pipe smoking: current, exclusive cigar and/or pipe smoking with previous history of cigarette smoking.

**Table 24:** **Current cigar smoking and aortic aneurysm by inhalation level and cigars per day**

**Study**	**Cohort name**	**Inhalation level**	**Cigars per day**	**Effect estimate**	**95% CI**	**Measure**	Primary/secondary*
**Shanks1998**	CPS-I		1 to 2	1.82	(1.11, 2.81)	IRR	Primary
**Shanks1998**	CPS-I		3 to 4	0.88	(0.35, 1.82)	IRR	Primary
**Shanks1998**	CPS-I		5+	2.62	(1.58, 4.09)	IRR	Primary
**Shanks1998**	CPS-I		1 to 2	3.03	(1.51, 5.43)	IRR	Secondary
**Shanks1998**	CPS-I		3 to 4	2.8	(1.34, 5.16)	IRR	Secondary
**Shanks1998**	CPS-I		5+	2.64	(1.26, 4.85)	IRR	Secondary
**Shanks1998**	CPS-I	None		1.73	(1.22, 2.39)	IRR	Primary
**Shanks1998**	CPS-I	Slight		1	(0.20, 2.92)	IRR	Primary
**Shanks1998**	CPS-I	Moderate-deep		4.94	(1.59, 11.52)	IRR	Primary
**Shanks1998**	CPS-I	None		2.18	(1.22, 3.59)	IRR	Secondary
**Shanks1998**	CPS-I	Slight		3.52	(1.69, 6.47)	IRR	Secondary
**Shanks1998**	CPS-I	Moderate-deep		2.94	(0.95, 6.87)	IRR	Secondary

*Primary cigar smoking: current, exclusive cigar smoking with no previous history of cigarette or pipe smoking; secondary cigar smoking: current, exclusive cigar smoking with previous history of cigarette or pipe smoking.

### COPD

Results for deaths from chronic obstructive pulmonary disease (COPD) are presented in Tables 25 and 26. Shanks and Burns found that COPD mortality was not associated with primary cigar smoking but was significantly associated with secondary cigar smoking (MR = 4.39, 95% CI = 3.02-6.16) in CPS-I (Table 25). Wald found that COPD mortality was not associated with primary cigar/pipe smoking but was significantly associated with secondary cigar/pipe smoking. Overall, current cigar smoking MRs ranged from 0.79 to 4.39, while current cigar/pipe smoking MRs ranged from 1.11 to 1.68. For cigars per day, there were no dose–response trends for primary cigar smoking, but there were strong dose–response trends for secondary cigar smoking in CPS-I (Table 26). For level of inhalation, there were suggestive dose–response trends for primary and secondary cigar smoking.

**Table 25:** **Current cigar smoking and chronic obstructive pulmonary disease (COPD)**

**Study**	**Cohort name**	**Sex**	**Cigar smoker deaths**	**Effect estimate**	**95% CI**	**Measure**	Primary/secondary*	**Adjustment**	**ICD codes**
***Current Cigar***									
**Kahn1966**	Dorn study		5	0.79	(0.25, 1.86)	SMR		Age	ICD 7: 500–502, 527.1
**Carstensen1987**	Swedish Census Cohort		1	1.3	(0.00, 7.45)	SMR		Age, residence	ICD 8: 490-492
**Lange1992**	Copenhagen City Heart Study	Men	22	3.7	(1.10, 12.00)	HR		Age	ICD 8: 490-492
**Lange1992**	Copenhagen City Heart Study	Women	10	10	(2.30, 48.00)	HR		Age	ICD 8: 490-492
**Shanks1998**	CPS-I		30	1.42	(0.96, 2.03)	IRR	Primary	Age	
**Shanks1998**	CPS-I		33	4.39	(3.02, 6.16)	IRR	Secondary	Age	
***Current Cigar and/or Pipe***									
**Wald1997**	British United Provident Association		40	1.11	(0.78, 1.59)	HR	Primary	Age	ICD 9: 416, 491–492, 496, 519
**Wald1997**	British United Provident Association		35	1.68	(1.16, 2.45)	HR	Secondary	Age	ICD 9: 416, 491–492, 496, 519

*Primary cigar smoking: current, exclusive cigar smoking with no previous history of cigarette or pipe smoking; primary cigar and/or pipe smoking: current, exclusive cigar and/or pipe smoking with no previous history of cigarette smoking; secondary cigar smoking: current, exclusive cigar smoking with previous history of cigarette or pipe smoking; secondary cigar and/or pipe smoking: current, exclusive cigar and/or pipe smoking with previous history of cigarette smoking.

**Table 26:** **Current cigar smoking and chronic obstructive pulmonary disease (COPD) by inhalation level and cigars per day**

**Study**	**Cohort name**	**Inhalation level**	**Cigars per day**	**Effect estimate**	**95% CI**	**Measure**	Primary/secondary*
**Shanks1998**	CPS-I		1 to 2	1.39	(0.74 2.38)	IRR	Primary
**Shanks1998**	CPS-I		3 to 4	1.78	(0.89 3.18)	IRR	Primary
**Shanks1998**	CPS-I		5+	1.03	(0.37 2.23)	IRR	Primary
**Shanks1998**	CPS-I		1 to 2	2.64	(1.06 5.44)	IRR	Secondary
**Shanks1998**	CPS-I		3 to 4	4.33	(2.07 7.97)	IRR	Secondary
**Shanks1998**	CPS-I		5+	6.68	(3.82 10.85)	IRR	Secondary
**Shanks1998**	CPS-I	None		1.09	(0.66 1.70)	IRR	Primary
**Shanks1998**	CPS-I	Slight		2.05	(0.66 4.77)	IRR	Primary
**Shanks1998**	CPS-I	Moderate-deep		4.52	(0.91 13.22)	IRR	Primary
**Shanks1998**	CPS-I	None		3.36	(1.96 5.39)	IRR	Secondary
**Shanks1998**	CPS-I	Slight		7.68	(3.31 15.14)	IRR	Secondary
**Shanks1998**	CPS-I	Moderate-deep		5.84	(2.34 12.02)	IRR	Secondary

*Primary cigar smoking: current, exclusive cigar smoking with no previous history of cigarette or pipe smoking; secondary cigar smoking: current, exclusive cigar smoking with previous history of cigarette or pipe smoking.

### Other causes of death

For a number of causes of death (atherosclerosis, cancers of the kidney, nasopharynx, colon and rectum), there were only single estimates of associations with cigar smoking (Table 27). All estimates come from the Dorn study cohort. Although estimates for colon and rectal cancer were also reported by Kahn, Heineman reported more up-to-date associations for these cancer sites. Only colon and rectal cancer were significantly associated with primary current cigar/pipe smoking after adjusting for age, calendar time, year of questionnaire response, SES, sedentary job. For colon cancer only, there was a significant dose-trend for the number of cigars smoked a day after adjusting for the same factors (p-trend = 0.004, data not shown).

**Table 27:** **Current cigar smoking and other causes of death**

**Study**	**Cohort name**	**Cause of death**	**Cigar smoker deaths**	**Effect estimate**	**95% CI**	**Measure**	Primary/secondary*	**Adjustment**	**ICD codes**
***Current Cigar***									
**Kahn1966**	Dorn study	Atherosclerosis	39	0.97	(0.69, 1.33)	SMR		Age	ICD7: 450
**Kahn1966**	Dorn study	Kidney cancer	6	0.77	(0.28, 1.69)	SMR		Age	ICD7: 180
***Current Cigar and/or Pipe***									
**Chow1993**	Dorn study	Nasopharyngeal carcinoma	2	1	(0.20, 5.20)	IRR	Primary	Age and calendar year	ICD7: 146
**Heineman1995**	Dorn study	Colon cancer	576	1.3	(1.10, 1.40)	IRR	Primary	Age, calendar time, year of questionnaire response, SES, sedentary job	ICD7: 153.0-153.3
**Heineman1995**	Dorn study	Rectal cancer	169	1.4	(1.20, 1.80)	IRR	Primary	Age, calendar time, year of questionnaire response, SES, sedentary job	ICD7: 154

*Primary cigar smoking: current, exclusive cigar smoking with no previous history of cigarette or pipe smoking; primary cigar and/or pipe smoking: current, exclusive cigar and/or pipe smoking with no previous history of cigarette smoking; secondary cigar smoking: current, exclusive cigar smoking with previous history of cigarette or pipe smoking; secondary cigar and/or pipe smoking: current, exclusive cigar and/or pipe smoking with previous history of cigarette smoking.

## Discussion

We have conducted a systematic review of the mortality risks associated with cigar smoking that identified 22 studies from 16 cohorts that assessed the relative mortality risks of cigar smokers compared with never tobacco users or never smokers. There was substantial variation across studies in terms of study characteristics including exposure definitions, types of effect measures, and adjustments for confounding. We prioritized estimates of primary cigar smoking (vs. never tobacco users or never smokers) with the aim of isolating the health effects of cigar smoking apart from other current or past tobacco smoking. We also placed emphasis on the larger cohorts, primarily the American Cancer Society’s Cancer Prevention Study I (CPS-I) and to a lesser extent CPS-II, which was conducted more recently than CPS-I but had half the number of primary cigar smokers as CPS-I (7,888 and 15,191 primary cigar smokers, respectively). The following mortality outcomes were significantly associated with primary cigar smoking overall in one or both CPS cohorts: all cause-mortality; oral cancer, esophageal cancer, pancreatic cancer, laryngeal cancer, lung cancer, coronary heart disease (CHD), and aortic aneurysm. Dose trends (cigars per day) for primary cigar smoking were observed for oral, esophageal, laryngeal, and lung cancers. Increased risks for oral, esophageal, laryngeal, and lung cancers were observed with increasing or any inhalation. It is notable that relative mortality risk was still highly elevated for oral, esophageal, and laryngeal cancer among primary cigar smokers reporting no inhalation, particularly in CPS-I which is consistent with the observation that cigar smoke is inhaled regardless of self-reported inhalation [40]. It is also notable that elevated risks of oral, esophageal, laryngeal cancers, and aortic aneurysm were observed among primary cigar smokers who smoked 1–2 cigars per day, although most of these risks did not reach statistical significance due to small sample size.

In CPS I, the risks were considerably higher for secondary cigar smokers than for primary cigar smokers for lung cancer (MRs = 6.29 and 2.1, respectively) and COPD (MRs = 4.39 and 1.42, respectively). As mentioned in the Introduction, the difference in risks could be due to differences in inhalation patterns between secondary cigar smokers and primary cigar smokers. Also, irreversible damage to the lungs due to past cigarette use can also explain the higher risks of lung cancer and COPD in secondary cigar smokers than in primary cigar smokers. These differences in risks between primary and secondary cigar smoking further reinforce the fact that exclusion of past cigarette smoking would better isolate the effects of cigar smoking for estimating disease risk.

Our results for mortality are consistent with recent studies of cigar smoking and incident cancers, such as those from the European Prospective Investigation into Cancer and Nutrition (EPIC) cohort and the International Head and Neck Cancer Epidemiology (INHANCE) Consortium. EPIC is a large multi-country cohort study of approximately half a million adults aged 35 to 70 years enrolled between 1991 and 1998 with a median of 9 years of follow up [41]. There were a total of 1,451 current (74%) or former (26%) cigar smokers who had never smoked cigarettes or pipes. Among the exclusive, current cigar smokers significantly elevated risks of lung (HR = 3.9) and upper aerodigestive tract (UADT) (oral cavity, larynx, esophagus excluding esophageal adenocarcinoma) (HR = 3.5) cancers as well as any tobacco-related cancer (HR = 1.6) were observed. The risk of combined lung/UADT/bladder cancer and all tobacco-related cancer increased with increasing inhalation level and increasing duration, but significantly elevated risk of any tobacco-related cancer was still observed when no inhalation was reported. Risk of lung/UADT/bladder cancer increased with increasing cigars smoked per day and was significantly increased across all cigar sizes (small, medium, and large). In a pooled analysis of 19 studies as part of the INHANCE Consortium (comprising 13,935 cases and 18,691 controls from 1981 to 2007), the associations between tobacco smoking (cigarette, cigar and pipe) and head and neck cancers (cancers of the oral cavity, pharynx, and larynx) were assessed [42]. Roughly one-third of the study participants were from the United States (35% of cases and 36% of controls), and most of the remaining subjects were from Latin America (28% of cases and 17% of controls) or Italy (18% of cases and 29% of controls). Among never cigarette smokers, the odds ratio (OR) for ever cigar smoking and head and neck cancers was significantly elevated (OR = 2.54, 95% CI = 1.93, 3.34), and increased with increasing frequency and duration of cigar smoking (p-trends ≤ 0.0001). At the same time, ORs for cigar and head and neck cancer were not elevated among ever cigarette smokers. In summary, the cigar associations from studies of incident disease are generally consistent with those of cause-specific mortality.

Our systematic review is subject to certain limitations. The studies included in this review were largely limited to study populations of white men in North America and Europe who smoked cigars in the 1960s or earlier. The predominant cigar type studied was the large cigar, whereas today, the US. cigar market consists of products manufactured with various shapes, sizes, tips, filters and packaging [3]. Limited data were available for a number of smoking-related cancers, including stomach, colorectal, and liver cancer. Finally, studies were often underpowered for detecting associations at the lowest levels of exposure.

Given the changes in cigar use patterns in the US and elsewhere since the 1960s, this review highlights the critical need for updated estimates of mortality risks due to cigar smoking. Additional studies that include women, non-whites, and younger adults in contemporary cohorts from the 1990s or later would better reflect current trends in cigar use. Studies that distinguish primary from secondary cigar smokers and exclude other current tobacco smokers would better isolate the effects of cigar smoking from other tobacco use. Data collection and analysis that include detailed information on the types of cigars typically used, the average number of cigars smoked per day, the depth of inhalation of cigar smoke, and the number of years smoking cigars would give a better sense of any dose–response relationship. Future studies incorporating biomarkers of tobacco exposure and potential harm such as cotinine and NNAL would better assess the levels of exposure to toxic constituents and intermediate health effects in cigar smokers, especially across cigar types and in comparison with cigarette smokers.

## Conclusions

In summary, cigar smoking carries many of the same health risks as cigarette smoking, which is consistent with the fact that the two products share similar levels of many of the same harmful constituents. Mortality risks from cigar smoking vary by level of exposure as measured by cigars per day and inhalation level. We have observed that some risks associated with cigar smoking can be as high or higher than those associated with cigarette smoking, especially at the highest doses and levels of inhalation for cigar smoking. However, even when no inhalation of cigar smoke is reported, risks of death from cancers of the upper aerodigestive tract (oral cavity, larynx, esophagus) are still highly elevated. This body of research would be strengthened by future studies that focus on primary cigar smoking, incorporate more contemporary and diverse study populations to better reflect the current patterns of cigar use in the US. Ideally, these studies would also collect detailed information on cigar type, level of exposure, and biomarkers of exposure and potential harm.

## Additional file

Additional file 1:
**Supplemental Web References.**
