# Supplementary material for: Systematic review of cigar smoking and all cause and smoking related mortality
Source: BMC Public Health. 2015 Apr 24;15:390. doi: 10.1186/s12889-015-1617-5 (PMC4408600; doi:10.1186/s12889-015-1617-5)
Supplement: Additional file 1: — Supplemental Web References. [file 12889_2015_1617_MOESM1_ESM.docx]

**Supplemental Web References**

w1. Hammond EC, Horn D: **Smoking and death rates. Report on forty-four months of follow-up of 187,783 men. I. Total mortality.** *JAMA* 1958, **166:**1159-1172.

w2. Hammond EC, Horn D: **Smoking and death rates: report on forty-four months of follow-up of 187,783 men. 2. Death rates by cause.** *JAMA* 1958, **166:**1294-1308.

w3. Gsell O: **Lung cancer and tobacco: The causes of lung cancer.** *Schweiz Med Wochenschr* 195, **81:**662-668.

w4. Nefzger MD, Quadfasel FA, Karl VC: **A retrospective study of smoking in Parkinson's disease.**  *Am J Epidemiol* 196, **88:**149-158.

w5. Pavan G, Salvadori B: **Mortality due to cancer in Venice from 1936 to 1946.** *Ospedale Maggiore* 1947, **35:**390-398.

w6. Doll R, Hill AB: **Smoking and carcinoma of the lung. Preliminary report.** *BMJ* 1950, **2:**739-748.

w7. Brummer P: **Coronary mortality and living standard. II. Coffee, tea, cocoa, alcohol and tobacco.** *Acta Med Scand* 1960, **186:**61-63.

w8. Wynder EL, Onderdonk J, Mantel N: **An epidemiological investigation of cancer of the bladder.** *Cancer* 1963, **16:**1388-1407.

w9. Lombard HL: **AN EPIDEMIOLOGICAL STUDY in LUNG CANCER.** *Cancer* 1965, **18:**1301-1309.

w10. Gsell O: **The epidemiology of bronchial carcinoma (environmental influences in Switzerland).** *Oncologia* 1965,1965-217.

w11. Hammond EG, Van Griethuysen TH, Dibeler JB, Sneddon AM, Halligan W: **Smoking habits and disease in New York state.** *New York State journal of medicine* 1965,764.

w12. Robinson P: **Smoking of burmese women during pregnancy and its influence on the mother, the fetus and the newborn (hebrew).** *Harefuah* 1965, **69:**37-39.

w13. Seidman H: **Lung cancer among Jewish, Catholic and Protestant males in New York city.** *Cancer* 1966, **19:**185-190.

w14. Oppers VM, Hoeflake GJ: **SMOKING, LUNG CANCER and COMPETITION of MORTALITY RATES.** *Tijdschrift voor Sociale Geneeskunde* 1966, **44:**2-12.

w15. Wynder EL, Shigematsu T: **Environmental factors of cancer of the colon and rectum.** *Cancer* 1967, **20:**1520-1561.

w16. Russell MH: **Smoking habits on the Far East Station in 1967.** *J Roy Nav Med Serv* 1969, **55:**76-83.

w17. De Haas JH: **Smoking patterns in the Netherlands. Are men going to smoke less?** *Hart Bulletin* 1973, **4:**77-86.

w18. Auerbach O, Garfinkel L, Hammond EC: **Relation of smoking and age to findings in lung parenchyma: a microscopic study.** *CHEST* 1974, **65:**29-35.

w19. Jensen OM: **Cancer of the lung and smoking in Danish women (Danish).** *Ugeskr Laeger* 1975, **137:**1489-1494.

w20. Nielsen PE, Krarup NB: **Consumption of tobacco in Denmark in the period 1920-1975 (Danish).**  *Ugeskr Laeger* 1976, **138:**2511-2516.

w21. Weiss W, Figueroa WG: **The characteristics of lung cancer due to chloromethyl ethers.** *J Occup Med* 1976, **18:**623-627.

w22. Goldbourt U, Medalie JH: **Characteristics of smokers, non-smokers and ex-smokers among 10,000 adult males in Israel. II. Physiologic, biochemical and genetic characteristics.** *Am J Epidemiol* 1977, **105:**75-86.

w23. Dupree EA, Meyer MB: **Role of risk factors in complications of diabetes mellitus.** *Am J Epidemiol* 1980, **112:**100-112.

w24. Wald NJ, Idle M, Boreham J, Bailey A: **Serum cotinine levels in pipe smokers: evidence against nicotine as cause of coronary heart disease.** *Lancet* 1981, **2:**775-777.

w25. Russell MAH: **Smoking, nicotine addiction and lung disease.** *European Journal of Respiratory Diseases* 1983, **62:**85-89.

w26. Jarvis M, West R, Tunstall-Pedoe H, Vesey C: **An evaluation of the intervention against smoking in the multiple risk factor intervention trial.** *Prev Med* 1984, **13:**501-509.

w27. Joly OG, Lubin JH, Caraballoso M: **Dark tobacco and lung cancer in Cuba.** *World Smoking Health* 1984, **9:**21-26.

w28. Repace JL, Lowrey AH: **A quantitative estimate of nonsmokers' lung cancer risk from passive smoking.** *Environment International* 1985, **11:**3-22.

w29. Bulpitt CJ, Beevers DG, Butler A, Coles EC, Hunt D, Munro-Faure AD, Newson RB, O'Riordan PW, Petrie JC, Rajagopalan B et al.: **The survival of treated hypertensive patients and their causes of death: a report from the DHSS hypertensive care computing project (DHCCP).** *J Hypertens* 1986, **4:**93-99.

w30. Anderson HR, Vallance P, Bland JM, Nohl F, Ebrahim S: **Prospective study of mortality associated with chronic lung disease and smoking in Papua New Guinea.** *Int J Epidemiol* 1988, **17:**56-61.

w31. Stellman SD, Boffetta P, Garfinkel L: **Smoking habits of 800,000 American men and women in relation to their occupations.** *Am J Ind Med* 1988, **13:**43-58.

w32. Roscoe RJ, Steenland K, Halperin WE, Beaumont JJ, Waxweiler RJ: **Lung cancer mortality among nonsmoking uranium miners exposed to radon daughters.** *JAMA* 1989, **262:**629-633.

w33. Sandler DP, Comstock GW, Helsing KJ, Shore DL: **Deaths from all causes in non-smokers who lived with smokers.** *Am J Public Health* 1989, **79:**163-167.

w34. Nyboe J, Jensen G, Appleyard M, Schnohr P: **Smoking and the Risk of First Acute Myocardial Infarction.** *Am Heart J* 1991, **122:**438-447.

w35. Shelley E, Daly L, Kilcoyne D, Graham I, Mulcahy R: **Risk factors for coronary heart disease: a population survey in County Kilkenny, Ireland, in 1985.** *Ir J Med Sci* 1991, **160:**22-28.

w36. Kunze E, Changclaude J, Frentzelbeyme R: **Life-Style and Occupational Risk-Factors for Bladder-Cancer in Germany - A Case Control Study.** *Cancer* 1992,1776-1790.

w37. Muscat JE, Wynder EL: **Tobacco, Alcohol, Asbestos, and Occupational Risk-Factors for Laryngeal-Cancer.** *Cancer* 1992,2244-2251.

w38. Egan KM, Gragoudas ES, Seddon JM, Walsh SM: **Smoking and the risk of early metastases from uveal melanoma.** *Ophthalmology* 1992, **99:**537-541.

w39. Hensrud DD, Sprafka JM: **The Smoking-Habits of Minnesota Physicians.** *Am J Public Health* 1993, **83:**415-417.

w40. Lavecchia C, Pagano R, Decarli A, Ferraroni M: **Smoking in Italy, 1990-1991.** *Tumori* 1994, **80:**175-180.

w41. Nelson DE, Davis RM, Chrismon JH, Giovino GA: **Pipe smoking in the United States, 1965-1991: prevalence and attributable mortality.** *Prev Med* 1996, **25:**91-99.

w42. **Cigar smoking among teenagers--United States, Massachusetts, and New York, 1996.** *MMWR* 1997, **46:**433-440.

w43. **Ingestion of cigarettes and cigarette butts by children--Rhode Island, January 1994-July 1996.** *MMWR* 1997, **46:**125-128.

w44. Sorahan T, Prior P, Lancashire RJ, Faux SP, Hulten MA, Peck IM, Stewart AM: **Childhood cancer and parental use of tobacco: Deaths from 1971 to 1976.** *Br J Cancer* 1997, **76:**1525-1531.

w45. Edwards R, Jakubovic M: **Effect on mortality of switching from cigarettes to pipes or cigars. "Switchers" will have had higher cumulative exposure to tobacco.** *BMJ* 1998, **316:**862-863.

w46. **Tobacco use among high school students--United States, 1997.** *MMWR* 1998, **47:**229-233.

w47. Gilpin EA, Pierce JP: **Cigar smoking in California: 1990-1996.** *Am J Prev Med* 1999, **16:**195-201.

w48. Jamner MS: **Cigar smoking among college students: Prevalence and correlates.** *Prev Med* 1999, **29:**187-194.

w49. **Tobacco use among middle and high school students--Florida, 1998 and 1999.** *MMWR* 1999, **48:**248-253.

w50. **State-specific prevalence of current cigarette and cigar smoking among adults--United States, 1998.** *MMWR* 1999, **48:**1034-1039.

w51. Grunbaum JA, Kann L, Kinchen SA, Ross JG, Gowda VR, Collins JL, Kolbe LJ: **Youth Risk Behavior Surveillance--National Alternative High School Youth Risk Behavior Survey, United States, 1998.** *MMWR* 1999, **48:**1-44.

w52. Boffetta P, Pershagen G, Jockel KH, Forastiere F, Gaborieau V, Heinrich J, Jahn I, Kreuzer M, Merletti F, Nyberg F et al.: **Cigar and pipe smoking and lung cancer risk: A multicenter study from Europe.** *J Natl Cancer Inst* 1999, **91:**697-701.

w53. Mannino DM, Gagnon RC, Petty TL, Lydick E: **Obstructive lung disease and low lung function in adults in the United States: data from the National Health and Nutrition Examination Survey, 1988-1994.** *Arch Intern Med*  2000,  **160:**1683-1689.

w54. **Youth tobacco surveillance--United States, 1998-1999.** *MMWR* 2000, **49:**1-94.

w55. Grunbaum JA, Kann L, Kinchen SA, Ross JG, Gowda VR, Collins JL, Kolbe LJ: **Youth risk behavior surveillance. National Alternative High School Youth Risk Behavior Survey, United States, 1998.** *J Sch Health* 2000, **70:**5-17.

w56. Pfeiffer W, Scholl J, Renz E, Cire L, Kentner M: **How healthy are company leaders? A cross-sectional study of the cardiovascular risk profile of managers.** *Arbeitsmedizin Sozialmedizin Umweltmedizin* 2001, **36:**126-131.

w57. **Youth tobacco surveillance--United States, 2000.** *MMWR* 2001, **50:**1-84.

w58. Pitard A, Brennan P, Clavel J, Greiser E, Lopez-Abente G, Chang-Claude J, Wahrendorf J, Serra C, Kogevinas M, Boffetta P: **Cigar, pipe, and cigarette smoking and bladder cancer risk in European men.** *Cancer Causes Control* 2001, **12:**551-556.

w59. Sharpe CR, Siemiatycki J: **Joint effects of smoking and body mass index on prostate cancer risk.** *Epidemiology* 2001, **12:**546-551.

w60. Woo JG, Pinney SM: **Retrospective smoking history data collection for deceased workers: completeness and accuracy of surrogate reports.** *J Occup Environ Med* 2002, **44:**915-923.

w61. **Tobacco use among middle and high school students--United States, 2002.** *MMWR* 2003, **52:**1096-1098.

w62. Henley SJ, Thun MJ, Chao A, Calle EE: **Association between exclusive pipe smoking and mortality from cancer and other diseases.** *J Natl Cancer Inst* 2004, **96:**853-861.

w63. Behrendt CE: **Mild and moderate-to-severe COPD in nonsmokers - Distinct demographic profiles.** *CHEST* 2005, **128:**1239-1244.

w64. Parducci DA, Puccetti M, Bianchi ML, Roselli MG, Vaghetti E, Settimi L, Orsi D, Battista G: **[Mortality among workers in a cigarette factory in Lucca (Tuscany)].** *Epidemiol Prev* 2005, **29:**271-277.

w65. Funck-Brentano C, Raphael M, Lafontaine M, Arnould JP, Verstuyft C, Lebot M, Costagliola D, Roussel R: **Effects of type of smoking (pipe, cigars or cigarettes) on biological indices of tobacco exposure and toxicity.** *Lung Cancer* 2006, **54:**11-18.

w66. **Tobacco use among adults--United States, 2005.** *MMWR* 2006, **55:**1145-1148.

w67. Marshall L, Schooley M, Ryan H, Cox P, Easton A, Healton C, Jackson K, Davis KC, Homsi G: **Youth tobacco surveillance--United States, 2001-2002.** *MMWR* 2006, **55:**1-56.

w68. Hassan MM, Abbruzzese JL, Bondy ML, Wolff RA, Vauthey JN, Pisters PW, Evans DB, Khan R, Lenzi R, Jiao L et al.: **Passive smoking and the use of noncigarette tobacco products in association with risk for pancreatic cancer: A case-control study.** *Cancer* 2007, **106:**2547-2556.

w69. Miller JW, Naimi TS, Brewer RD, Jones SE: **Binge drinking and associated health risk behaviors among high school students.** *Pediatrics* 2007,  **119:**76-85.

w70. Randi G, Scotti L, Bosetti C, Talamini R, Negri E, Levi F, Franceschis S, La VC: **Pipe smoking and cancers of the upper digestive tract.** *Int J Cancer* 2007, **121:**2049-2051.

w71. Shugars DC, Adesanya M, Diehl SR, Redman RS, Malley KJ, Silva RG, Farish SE, Francis T, Craig RM, Jones-Richardson T et al.: **Self-awareness of premalignant oral lesions among veterans attending six U.S. Veterans Affairs Medical Centers.** *Spec Care Dentist* 2007, **27:**87-94.

w72. Rohrmann S, Genkinger JM, Burke A, Helzlsouer KJ, Comstock GW, Alberg AJ, Platz EA: **Smoking and risk of fatal prostate cancer in a prospective U.S. study.** *Urology* 2007, **69:**721-725.

w73. Gruskin EP, Greenwood GL, Matevia M, Pollack LM, Bye LL, Albright V: **Cigar and smokeless tobacco use in the lesbian, gay, and bisexual population.** *Nicotine Tob Res* 2007, **9:**937-940.

w74. O'Connor RJ, McNeill A, Borland R, Hammond D, King B, Boudreau C, Cummings KM: **Smokers' beliefs about the relative safety of other tobacco products: Findings from the ITC Collaboration.** *Nicotine Tob Res* 2007, **9:**1033-1042.

w75. Streppel MT, Boshuizen HC, Ocke MC, Kok FJ, Kromhout D: **Mortality and life expectancy in relation to long-term cigarette, cigar and pipe smoking: the Zutphen Study.** *Tob Control* 2007, **16:**107-113.

w76. Boffetta P: **Tobacco smoking and risk of bladder cancer.** *Scand J Urol Nephrol Suppl* 2008,45-54.

w77. Mulvaney C, Kendrick D, Towner E, Brussoni M, Hayes M, Powell J, Robertson S, Ward H: **Fatal and non-fatal fire injuries in England 1995-2004: time trends and inequalities by age, sex and area deprivation.** *J Public Health (Oxf )* 2009, **31:**154-161.

w78. Bell RA, Arcury TA, Chen H, Anderson AM, Savoca MR, Kohrman T, Quandt SA: **Use of tobacco products among rural older adults: prevalence of ever use and cumulative lifetime use.** *Addict Behav* 2009, **34:**662-667.

w79. Yeo TP, Hruban RH, Brody J, Brune K, Fitzgerald S, Yeo CJ: **Assessment of "gene-environment" interaction in cases of familial and sporadic pancreatic cancer.** *J Gastrointest Surg* 2009, **13:**1487-1494.

w80. McCormack VA, Agudo A, Dahm CC, Overvad K, Olsen A, Tjonneland A, Kaaks R, Boeing H, Manjer J, Almquist M et al.: **Cigar and pipe smoking and cancer risk in the European Prospective Investigation into Cancer and Nutrition (EPIC).** *Int J Cancer* 2010, **127:**2402-2411.

w81. Rodriguez J, Jiang R, Johnson WC, MacKenzie BA, Smith LJ, Barr RG: **The Association of Pipe and Cigar Use With Cotinine Levels, Lung Function, and Airflow Obstruction A Cross-sectional Study.** *Ann Intern Med* 2010, **152:**201-U17.

w82. Tilloy E, Cottel D, Ruidavets JB, Arveiler D, Ducimetiere P, Bongard V, Haas B, Ferrieres J, Wagner A, Bingham A et al.: **Characteristics of current smokers, former smokers, and second-hand exposure and evolution between 1985 and 2007.** *Eur J Cardiovasc Prev Rehabil* 2010, **17:**730-736.

w83. Champagne BM, Sebrie EM, Schargrodsky H, Pramparo P, Boissonnet C, Wilson E: **Tobacco smoking in seven Latin American cities: the CARMELA study.** *Tob Control* 2010, **19:**457-462.

w84. Krahn L, Slocumb N, Silber M: **Injuries and property damage due to smoking in narcoleptic patients.** *Journal of Sleep Research* 2010, **19:**148.

w85. **Any tobacco use in 13 States --- behavioral risk factor surveillance system, 2008.** *MMWR* 2010, **59:**946-950.

w86. McClave AK, Whitney N, Thorne SL, Mariolis P, Dube SR, Engstrom M: **Adult tobacco survey - 19 States, 2003-2007.** *MMWR* 2010, **59:**1-75.

w87. Peykari NF, Tehrani FR, Afzali HM, Dovvon MR, Djalalinia SS: **Smoking habits among Iranian general practitioners.** *J Egypt Public Health Assoc* 2010, **85:**97-112.

w88. Carroll WR, Foushee HR, Hardy CM, Floyd T, Sinclair CF, Scarinci I: **Tobacco use among rural African American young adult males.** *Otolaryngol Head Neck Surg* 2011, **145:**259-263.

w89. Olmsted KLR, Bray RM, Guzman CMR, Williams J, Kruger H: **Overlap in Use of Different Types of Tobacco Among Active Duty Military Personnel.** *Nicotine Tob Res* 2011, **13:**691-698.

w90. Blazer DG, Wu LT: **Patterns of tobacco use and tobacco-related psychiatric morbidity and substance use among middle-aged and older adults in the United States.** *Aging & Mental Health* 2012, **16:**296-304.

w91. Bhan N, Srivastava S, Agrawal S, Subramanyam M, Millett C, Selvaraj S, Subramanian SV: **Are socioeconomic disparities in tobacco consumption increasing in India? A repeated cross-sectional multilevel analysis
7
8.** *Bmj Open* 2012, **2**.

w92. Chue AL, Carrara VI, Paw MK, Pimanpanarak M, Wiladphaingern J, van Vugt M, Lee SJ, Nosten F, McGready R: **Is areca innocent? The effect of areca (betel) nut chewing in a population of pregnant women on the Thai-Myanmar border
5
10.** *International Health* 2012, **4:**204-209.

w93. Kurtoglu E, Akturk E, Korkmaz H, Sincer I, Yilmaz M, Erdem K, Celik A, Ozdemir R: **Elevated red blood cell distribution width in healthy smokers
7.** *Turk Kardiyol Dernegi Ars* 2013, **41:**199-206.

w94. Tami-Maury I, Vidrine DJ, Fletcher FE, Danysh H, Arduino R, Gritz ER: **Poly-Tobacco Use Among HIV-Positive Smokers: Implications for Smoking Cessation Efforts
2
14.** *Nicotine & Tobacco Research* 2013, **15:**2100-2106.

w95. Wu LT, Swartz MS, Burchett B, Blazer DG: **Tobacco use among Asian Americans, Native Hawaiians/Pacific Islanders, and mixed-race individuals: 2002-2010
3
15.** *Drug and Alcohol Dependence* 2013, **132:**87-94.

w96. Abughosh S, Wu IH, Wang X, Essien EJ, Peters RJ, Almogbel YS, Sansgiry SS: **Predictors of cigarettes smoking among adults in five countries: China, Jordan, India, Taiwan, and Saudi Arabia
1.** *Value Health* 2014, **17:**A172.

w97. Grossman E, Duenas MI, Colucci A, Fruchter R, Wang B: **Effectiveness of using non-clinicians in delivering a brief smoking cessation intervention in the emergency department
2.** *J Gen Intern Med* 2014, **29:**S81.

w98. Kim C, Chapman RS, Hu W, He XZ, Hosgood HD, Liu LZ, Lai H, Chen W, Silverman DT, Vermeulen R et al.: **Smoky coal, tobacco smoking, and lung cancer risk in Xuanwei, China
1
11.** *Lung Cancer* 2014, **84:**31-35.

w99. Hickey N, Mulcahy R, Daly L, Graham I, O'Donoghue S, Kennedy C: **Cigar and pipe smoking related to four year survival of coronary patients.** *Br Heart J* 1983, **49:**423-426.

w100. Iribarren C, Tekawa IS, Sidney S, Friedman GD: **Effect of cigar smoking on the risk of cardiovascular disease, chronic obstructive pulmonary disease, and cancer in men.** *N Engl J Med* 1999, **340:**1773-1780.

w101. Mateen FJ, Carone M, Alam N, Streatfield PK, Black RE: **A population-based case-control study of 1250 stroke deaths in rural Bangladesh.** *Eur J Neurol* 2012, **19:**999-1006.

w102. Gyntelberg F, Lauridsen L, Pedersen PB, Schubell K: **Smoking and risk of myocardial infarction in Copenhagen men aged 40-59 with special reference to cheroot smoking.** *Lancet* 1981, **1:**987-989.

w103. Matroos A, Magnus K, Strackee J: **Fatal and nonfatal coronary attacks in relation to smoking in some Dutch communities.** *Am J Epidemiol* 1979, **109:**145-151.

w104. Higgins ITT, Mahan CM, Wynder EL: **Lung cancer among cigar and pipe smokers.** *Prev Med* 1988, **17:**116-128.

w105. Hein HO, Suadicani P, Gyntelberg F: **Ischaemic heart disease incidence by social class and form of smoking: the Copenhagen Male Study--17 years' follow-up.** *J Intern Med* 1992, **231:**477-483.

w106. Gupta PC, Mehta FS, Pindborg JJ: **Mortality among reverse chutta smokers in South India.** *BMJ* 1984, **289:**865-866.

w107. Joly OG, Lubin JH, Caraballoso M: **Dark tobacco and lung cancer in Cuba.** *J Natl Cancer Inst* 1983, **70:**1033-1039.

w108. Hurt RD, Offord KP, Croghan IT, Gomez-Dahl L, Kottke TE, Morse RM, Melton III LJ: **Mortality following inpatient addictions treatment: Role of tobacco use in a community-based cohort.** *JAMA* 1996, **275:**1097-1103.

w109. Zahm SH, Heineman EF, Vaught JB: **Soft tissue sarcoma and tobacco use: Data from a prospective cohort study of United States veterans.** *Cancer Causes and Control* 1992, **3:**371-376.

w110. Fernberg P, Odenbro A, Bellocco R, Boffetta P, Pawitan Y, Adami J: **Tobacco use, body mass index and the risk of malignant lymphomas--a nationwide cohort study in Sweden.**  *Int J Cancer* 2006, **118:**2298-2302.

w111. Strobel M: **Mortality in relation to smoking.** *Helvetica Meidca Acta* 1965, **32:**623-630.

w112. Abelin T: **Lung cancer and smoking in switzerland. An analysis of the smoking habits in all fatal cases of lung cancer in switzerland, 1951-1960.** *Schweiz Med Wochenschr* 1965, **95:**253-259.

w113. Gsell O, Strobel M: **MALIGNANT TUMORS and THEIR RELATIONSHIP to SMOKING. OBSERVATIONS on SWISS.** *Oncologia* 1966,1966-51.

w114. Staszewski J: **Lung cancer mortality, smoking and atmospheric pollution in Poland.** *Przeglad Epidemiologiczny* 1968, **22:**131-138.

w115. Abelin T, Gsell O: **[Cigar and pipe smoking as contributory factors in lung cancer, coronary disease, and total mortality].** *Schweiz Med Wochenschr* 1974, **Aug.3:**1098-1103.

w116. Furberg C, Isacsson SO: **Stopping smoking prolongs life (Swedish).** *Lakartidningen* 1975, **72:**2936-2938.

w117. Holme I: **Mortality in Norwegian physicians in relation to smoking habits. A 20 year follow up (Norwegian).** *Tidsskrift for den Norske Laegeforening* 1976, **96:**620-627.

w118. Gsell O, Abelin T, Wieltschnig E: **[Smoking and mortality in Swiss physicians: results of an 18-year survey].** *Bull Schweiz Akad Med Wiss* 1979, **35:**71-82.

w119. Magnus K, Matroos A, Strackee J: **Relationship between smoking habits, physical activity and coronary disease (Zeist project). I. Smoking habits.** *Ned Tijdschr Geneeskd* 1980, **124:**1445-1449.

w120. Hein HO, Suadicani P, Gyntelberg F: **[Social inequalities as a risk of ischemic heart disease--a matter of smoking habits? 17 years' follow-up in the Copenhagen Male Study].** *Ugeskr Laeger* 1993, **155:**1935-1939.

w121. Lange P, Nyboe J, Appleyard M, Jensen G, Schnohr P: **[Tobacco, lung cancer and chronic obstructive lung disease. Results from the Osterbro study].** *Ugeskr Laeger* 1993, **155:**2333-2337.

w122. Fan Z, Li F, Wang Z, Huang Z, Yi Y, Zhang Z, Yang Z, Zhang H, Ma Y, Zen X: **[A cohort study on the relationship between the mortality of cerebro-vascular diseases and farmer smokers].** *Hua Xi Yi Ke Da Xue Xue Bao 1994 Sep* 1994, **25:**349-352.

w123. Fan Z, Li F, Yi Y, Wang Z, Huang Z, Zhang X, Yang S, Jiang Y, Zhang Y, Zhang H et al.: **[A cohort study on the relationship between standardized mortality of pulmonary heart disease and smoking].** *Hua Xi Yi Ke Da Xue Xue Bao* 1996, **27:**199-202.

w124. Simmonds FAH, Davies TW, MacDonald N:  **Smoking and chest disease. Report of the joint tuberculosis council of Great Britain.** *Tubercle* 1960, **41:**290-301.

w125. Delarue NC: **A review of some important problems concerning lung cancer. I. Considerations of epidemiology, etiology and pathogenesis.** *Can Med Assoc J* 1961, **84:**1374-1385.

w126. Rot A: **Smoking and lung cancer.** *Krebsarzt* 1962, **17:**97-102.

w127. the annual incidence of cancer of the lung: **the annual incidence of cancer of the lung.** *Danish Medical Bulletin* 1962, **9:**100-102.

w128. Homburger F, Treger A, Baker JR: **Mouse-skin painting with smoke condensates from cigarettes made of pipe, cigar, and cigarette tobaccos.** *J Nat Cancer Inst* 1963, **31:**1445-1459.

w129. Weiss W, Weiss WA: **Effect of tobacco smoke solutions on paramecium.** *Arch Environ Health* 1964, **9:**500-504.

w130. Michaels L: **Aetiology of coronary artery disease: An historical approach.** *Br Heart J* 1966, **28:**258-264.

w131. Louw JH: **Resection and end-to-end anastomosis in the management of atresia and stenosis of the small bowel.** *Surgery* 1967, **62:**940-950.

w132. Small JD: **Fatal enteroeolitis in hamsters given lincomyctn hydroehloride -.** *Lab Anim Care* 1968, **18:**411-420.

w133. Garfinkel L: **The association between cigarette smoking and coronary heart disease and other vascular diseases.** *Bull N Acad Med* 1968, **44:**1495-1501.

w134. Bell JAE: **Mortality rates of smokers.** *Canad Med Ass J* 1969, **101:**402-416.

w135. Bell JAE: **Mortality rates of smokers.** *Canad Med Ass J* 1969, **101:**362-363.

w136. **The smoking disease.** *Brit Med J* 1970, **1:**61-62.

w137. Cowie J, Sillett RW, Ball K: **Carbon-monoxide absorption by cigarette smokers who change to smoking cigars.** *Lancet* 1973, **1:**1033-1035.

w138. Castleden CM, Cole PV: **Inhalation of tobacco smoke by pipe and cigar smokers.** *Lancet* 1973, **2:**21-22.

w139. Brown CT: **Freud and cancer.** *Tex Med* 1974, **70:**62-64.

w140. Lough J: **Cigarette smoking, coronary heart disease and sudden death.** *Can Med Assoc J* 1975, **113:**919.

w141. Gsell O: **Smoking and lung cancer in Switzerland 1900-1973.** *Praxis* 1975, **64:**643-650.

w142. Hoppe R: **An analysis of 20,000 cases of suspected lung cancer.** *Praxis und Klinik der Pneumologie* 1977, **31:**872-884.

w143. Berson SD, Brandt FA: **Primary pulmonary sporotrichosis with unusual fungal morphology.** *Thorax* 1977, **32:**505-508.

w144. Shoenfeld Y: **Smoking and heart diseas.** *Family Physician* 1977,  **6:**283-290+113.

w145. Brodsky BF, Zakharchuk Y: **Treatment of appendicular peritonitis in children.** *Khirurgiya* 1979, **55:**32-34.

w146. Elson LA, Betts TE: **Death rates from cancer of the respiratory and oral tracts in different countries, in relation to the types of tobacco smoked.** *Eur J Cancer* 1981, **17:**109-113.

w147. DeMarini DM: **Genotoxicity of tobacco smoke and tobacco smoke condensate.** *Mutation Research* 1983, **114:**59-89.

w148. Dayal H, Kinman J: **Epidemiology of kidney cancer.** *Semin Oncol* 1983, **10:**366-377.

w149. Egsmose T: **The tobacco problem in Denmark and the difficulties in implementing an organized plan of action.** *Tokai J Exp Clin Med* 1985, **10:**457-463.

w150. Baden E: **Prevention of cancer of the oral cavity and pharynx.** *CA Cancer J Clin* 1987, **37:**49-62.

w151. Hahn R, Ewers U, Jermann E, Freier I, Brockhaus A, Schlipkoter HW: **Cadmium in kidney cortex of inhabitants of North-West Germany: its relationship to age, sex, smoking and environmental pollution by cadmium.** *Int Arch Occup Environ Health* 1987, **59:**165-176.

w152. Wilhelmsen L: **Coronary heart disease: epidemiology of smoking and intervention studies of smoking.** *Am Heart J* 1988, **115:**242-249.

w153. **Honduras.**  *Backgr Notes Ser* 1989, **Sep.:**1-7.

w154. Antolin I, Uria H, Tolivia D, Rodriguezcolunga MJ, Rodriguez C, Kotler ML, Menendezpelaez A: **Porphyrin Accumulation in the Harderian Glands of Female Syrian-Hamster Results in Mitochondrial Damage and Cell-Death.** *Anatomical Record* 1994, **239:**349-359.

w155. Giovino GA, Schooley MW, Zhu BP, Chrismon JH, Tomar SL, Peddicord JP, Merritt RK, Husten CG, Eriksen MP: **Surveillance for selected tobacco-use behaviors--United States, 1900-1994.** *MMWR* 1994, **18:**1-43.

w156. Shopland DR: **Tobacco use and its contribution to early cancer mortality with a special emphasis on cigarette smoking.** *Environ Health Perspect* 1995, **103:**131-142.

w157. **[Is smoking pipes or cigars harmful?].** *Ned Tijdschr Geneeskd* 1996, **140:**1700.

w158. Knol K: **[Is smoking cigars or pipes harmful?].** *Ned Tijdschr Geneeskd* 1997, **141:**547-548.

w159. Hernandez-Avila M: **About 5.2 trillions of cigarettes are consumed in the world in a year.**  *Salud publica de Mexico* 1997, **39:**495-496.

w160. McGuirt WF: **Cigar smoking.** *Otolaryngology-Head and Neck Surgery* 1998, **119:**151-152.

w161. Lubin JH, Fraumeni JF: **Effect on mortality of switching from cigarettes to pipes or cigars. American study supported conclusions.** *BMJ* 1998, **316:**863-864.

w162. O'Driscoll BR: **Effect on mortality of switching from cigarettes to pipes or cigars. Study underestimated difference in risk.** *BMJ* 1998, **316:**862.

w163. O'Driscoll BR, Jarvis MJ, Edwards R, Jakubovic M, Lubin JH, Fraumeni JF, Wald NJ, Watt HC: **Effect on mortality of switching from cigarettes to pipes or cigars (multiple letters) [5].** *BMJ* 1998, **316:**862-864.

w164. Wald NJ, Watt HC: **Effect on mortality of switching from cigarettes to pipes or cigars - American study supported conclusions - Authors' reply.** *BMJ* 1998, **316:**863-864.

w165. Doll R: **Uncovering the effects of smoking: historical perspective.** *Stat Methods Med Res* 1998, **7:**87-117.

w166. Jarvis MJ: **Effect on mortality of switching from cigarettes to pipes or cigars. Patterns of inhalation are important.** *BMJ* 1998, **316:**862-864.

w167. Dahal G, Gauhl F, Pasberg-Gauhl C, Hughes JD, Thottappilly G, Lockhart BEL: **Evaluation of micropropagated plantain and banana (Musa spp.) for banana streak badnavirus incidence under field and screenhouse conditions in Nigeria.** *Annals of Applied Biology* 1999, **134:**181-191.

w168. Bell E: **The dud cigar? - Cochrane collaboration and the saga of human albumin.** *Adverse Drug React Toxicol Rev* 1999, **18:**149-163.

w169. Foley GV: **Cigars: Danger ahead.** *Cancer Practice* 1999, **7:**54.

w170. Baker F, Ainsworth SR, Dye JT, Crammer C, Thun MJ, Hoffmann D, Repace JL, Henningfield JE, Slade J, Pinney J et al.: **Health risks associated with cigar smoking.** *JAMA* 2000, **284:**735-740.

w171. Larimore WL, Cline MK: **Keeping normal labor normal.** *Prim Care* 2000, **27:**221-236.

w172. Hecht SS, Hoffmann D: **Re: Cigar smoking in men and risk of death from tobacco-related cancers.** *J Natl Cancer Inst* 2000, **92:**2040.

w173. DeLucia AJ: **Tobacco abuse and its treatment. Turning old and new issues into opportunities for the occupational health nurse.** *AAOHN J* 2001, **49:**243-259.

w174. Tsokos M, Longauer F, Kardosova V, Gavel A, Anders S, Schulz F: **Maternal death in pregnancy from HELLP syndrome. A report of three medico-legal autopsy cases with special reference to distinctive histopathological alterations.** *Int J Legal Med* 2002, **116:**50-53.

w175. Bromen K, Jockel K: **Epidemiology of lung cancer.** *Atemwegs - und Lungenkrankheiten* 2002, **28:**524-533.

w176. McDonald LJ, Bhatia RS, Hollett PD: **Deposition of cigar smoke particles in the lung: Evaluation with ventilation scan using Tc-99m-Labeled sulfur colloid particles.** *J Nucl Med* 2002, **43:**1591-1595.

w177. Horsey PJ: **The albumin meta-analysis from the Cochrane Goup - Egg on whose face?** *British Journal of Intensive Care* 2002, **12:**54-57.

w178. Rachbauer F, Mangat J, Bodner G, Eichberger P, Krismer M: **Heat distribution and heat transport in bone during radiofrequency catheter ablation.** *Arch Orthop Trauma Surg* 2003, **123:**86-90.

w179. Fukunaga M: **Neuron-specific enolase-producing leiomyosarcoma of the mesentery: Case report.** *APMIS* 2004, **112:**105-108.

w180. Pascual PF, Vicens LS: **Historic, social and economic aspects.** *Adicciones* 2004, **16:**13-24.

w181. Sasco AJ, Secretan MB, Straif K: **Tobacco smoking and cancer: A brief review of recent epidemiological evidence.** *Lung Cancer* 2004, **45:**S3-S9.

w182. Tanski SE, Prokhorov AV, Klein JD: **Youth and tobacco.** *Minerva Pediatr* 2004, **56:**553-565.

w183. Ishida M, Shiomi H, Naka S, Tani T, Okabe H: **Leiomyoma of the gallbladder in a patient with metastatic gastrointestinal stromal tumor in the liver: A case report with differential diagnostic considerations.** *Oncology Letters* 2004,1171-1173.

w184. Adeyemo WL: **Sigmund Freud: smoking habit, oral cancer and euthanasia.** *Niger J Med* 2004, **13:**189-195.

w185. Symm B, Vazquez MM, Blackshear Y, Tinsley S: **Cigar smoking: An ignored public health threat.** *J Prim Prev* 2005, **26:**363-375.

w186. Streppel MT, Ocke MC, Boshuizen HC, Kok FJ, Kromhout D: **Mortality due to long-term cigarette, cigar and pipe smoking. The Zutphen Study.** *European Journal of Epidemiology* 2006, **21:**71-72.

w187. Padmaja IJ, Ramani TV, Kalyani S: **Cutaneous zygomycosis: Necrotising fascitis due to Saksenaea vasiformis.** *Indian J Med Microbiol* 2006, **24:**58-60.

w188. Laugesen M: **Snuffing out cigarette sales and the smoking deaths epidemic.** *N Z Med J* 2007, **120:**U2587.

w189. Cafarchia C, Sasanelli M, Lia RP, De CD, Guillot J, Otranto D: **Lymphocutaneous and nasal sporotrichosis in a dog from southern Italy: Case report.** *Mycopathologia* 2007, **163:**75-79.

w190. Viegas CADA: **Noncigarette forms of tobacco use.** *J Bras Pneumol* 2008, **34:**1069-1073.

w191. Lierke EG, Hemsel T, Leibenger M: **Local ultrasonic hyperthermia and thermo-ablation: A description and theoretical evaluation of two alternative concepts for the heat therapy of tumours.** *Acta Acustica United with Acustica* 2008, **94:**369-381.

w192. Best D, Binns HJ, Forman JA, Karr CJ, Paulson JA, Osterhoudt KC, Roberts JR, Sandel MT, Seltzer JM, Wright RO et al.: **Technical report - Secondhand and prenatal tobacco smoke exposure.** *Pediatrics* 2009, **124:**e1017-e1044.

w193. Palmiere C, Staub C, La HR, Mangin P:  **Ignition of a human body by a modest external source: A case report.** *Forensic Science International* 2009, **188:**e17-e19.

w194. Oechsler S, Zimmer G, Pedal I, Skopp G: **Has the transdermal patch gone up in smoke? A fatal fentanyl intoxication.** *Arch Kriminol* 2009, **224:**26-35.

w195. Ward RJ, Clements KD, Choat JH, Angert ER: **Cytology of terminally differentiated Epulopiscium mother cells.** *DNA Cell Biol* 2009, **28:**57-64.

w196. Buchan BW, Ledeboer NA: **Identification of Two Borderline Oxacillin-Resistant Strains of Staphylococcus aureus From Routine Nares Swab Specimens by One of Three Chromogenic Agars Evaluated for the Detection of MRSA.** *Am J Clin Pathol* 2010, **134:**921-927.

w197. Steinberg MB: **Tobacco Smoke by Any Other Name Is Still as Deadly.** *Ann Intern Med* 2010, **152:**259-U98.

w198. Bergeron C: **Cytological characteristics of glandular lesions and their differential diagnosis.** *Virchows Archiv* 2010, **457:**136.

w199. Da Rocha Azevedo AL, Martins CJ, Lima RB, Sakuma TH: **Oral manifestations of disseminated sporotrichosis in a patient with AIDS.** *Journal of the American Academy of Dermatology* 2010, **62:**AB83.

w200. Thomas K, Salafia CM, Buhimschi I, Buhimschi CS, Zambrano E, Sottile M: **Reliability of automated neutrophil quantitation in digitized H&e stained slides: Pilot analysis of correlation with amniotic fluid proteomics score.** *Pediatric and Developmental Pathology* 2010, **13:**149-150.

w201. Koukourakis G, Zacharias G: **Nicotine has implications in different tumors types. Expert's eye making a literature analysis.** *J BUON* 2011, **16:**210-214.

w202. Solomon SB: **Expanded applications of focused ultrasound.** *CardioVascular and Interventional Radiology* 2011, **34:**443.

w203. Agale S, Grover S, Zode R, Hande S: **Primary cutaneous leiomysarcoma.** *Indian Journal of Dermatology* 2011, **56:**728-730.

w204. Krahn L, Slocumb N, Silber M: **Injuries and property damage due to smoking in narcoleptic patients.** *Sleep and Biological Rhythms* 2011, **9:**311-312.

w205. Carlile R, Kerman D, Bhamidimarri K: **A probable case of hereditary hemorrhagic telangiectasia with recurrent massive gastrointestinal bleeding requiring hemicolectomy despite medical management.** *American Journal of Gastroenterology* 2012, **107:**S482-S483.

w206. Jeon JY, Kim MJ, Lee JS, Kim I: **Fulminant emphysematous pancreatitis.** *HPB* 2012, **1914:**619-620.

w207. Ramareddy RS, Alladi A, Siddapa OS, Deepti V, Akthar T, Mamata B: **Surgical complications of Ascaris lumbricoides in children.** *J Indian Assoc Pediatr Surg* 2012, **17:**116-119.

w208. Letaiova S, Medveova A, Ovikova A, Duinska M, Volkovova K, Mosoiu C, Bartonova A: **Bladder cancer, a review of the environmental risk factors.** *Environmental Health* 2012, **11:**S11.

w209. Bai C: **Keynote: Epidemiology and burden of smoking related diseases in china
4.** *J Thorac Oncol* 2013, **8:**S128.

w210. Katsiki N, Papadopoulou SK, Fachantidou AI, Mikhailidis DP: **Smoking and vascular risk: Are all forms of smoking harmful to all types of vascular disease?
6.** *Public Health* 2013, **127:**435-441.

w211. Kedar P: **Red cell membrane pathology in hereditary spherocytosis in India
3.** *Indian J Hematol Blood Transfus* 2013, **29:**245-246.

w212. Rothman KJ, Gallacher JEJ, Hatch EE: **Rebuttal: When it comes to scientific inference, sometimes a cigar is just a cigar
5.** *Int J Epidemiol* 2013, **42:**1026-1028.

w213. Gsell OR, Abelin R: **Cigar and pipe smoking in relation to lung cancer and excess mortality.** *J Natl Cancer Inst* 1972, **48:**1795-1803.

w214. Hsing AW, McLaughlin JK, Hrubec Z, Blot WJ, Fraumeni JF: **Tobacco Use and Prostate-Cancer - 26-Year Follow-Up of United-States Veterans.** *Am J Epidemiol* 1991, **133:**437-441.

w215. Heineman EF, Zahm SH, McLaughlin JK, Vaught JB, Hrubec Z: **A prospective study of tobacco use and multiple myeloma: Evidence against an association.** *Cancer Causes and Control* 1992, **3:**31-36.

w216. Schroll M: **Smoking habits in the Glostrup population of men and women, born in 1914. Implications for health, evaluated from ten-year mortality, Incidence of cardiovascular manifestations and pulmonary function, 1964--1974.** *Acta Med Scand* 1980, **208:**245-256.

w217. Doyle JT, Dawber TR, Kannel WB, Kinch SH, Kahn HA: **The relationship of cigarette smoking to coronary heart disease. The second report of the combined experience of the Albany, N.Y., and Framingham, Mass., studies.** *JAMA* 1964, **190:**886-890.

w218. Hammond EG, Horn D: **The relationship between human smoking habits and death rates.** *JAMA* 1954, **155:**1316-1328.

w219. Hammond EC, Horn D: **Lung cancer death rates in relation to smoking.** *C A* 1958, **8:**42-43.

w220. Dorn HF: **TOBACCO CONSUMPTION and MORTALITY from CANCER and OTHER DISEASES -.** *Public health reports* 1959, **74:**581-593.

w221. Doyle JT, Dawber TR, Kannel WB, Heslin AS, Kahn HA: **Cigarette smoking and coronary heart disease. Combined experience of the Albany and Framingham studies.** *N Engl J Med* 1962, **266:**796-801.

w222. Hammond EC: **Smoking in relation to mortality and morbidity. Findings in first thirty-four months of follow-up in a prospective study started in 1959.** *J Natl Cancer Inst* 1964, **32:**1161-1188.

w223. Bell JA, Laing DH: **Statistical analysis of mortality rates of cigarette, pipe and cigar smokers.** *Can Med Assoc J* 1969, **100:**806-810.

w224. Rogot E: **Smoking and mortality among U.S. veterans.** *J Chronic Dis* 1974, **27:**189-203.

w225. Iribarren C, Sidney S, Tekawa I, Friedman GD, Permanente K: **Impact of cigar smoking on coronary, other heart-circulatory, cancer, and total mortality among never cigarette and never pipe smoking men.** *Circulation* 1998, **97:**822.

w226. Jacobs EJ, Shapiro JA, Thun MJ: **Cigar smoking in men and risk of death from tobacco-related cancers.** *Am J Epidemiol* 1999, **149:**S71.

w227. Lopez-Jimenez F, Sesso HD, Gaziano M:  **Cigar smoking and the risk of total and cardiovascular mortality in men.** *Circulation* 2001, **104:**794.
